# Identification of the shared genes in type 2 diabetes mellitus and osteoarthritis and the role of quercetin

**DOI:** 10.1111/jcmm.18127

**Published:** 2024-02-08

**Authors:** Siyuan Song, Jiangyi Yu

**Affiliations:** ^1^ Affiliated Hospital of Nanjing University of Chinese Medicine Nanjing Jiangsu China; ^2^ Nanjing University of Chinese Medicine Nanjing Jiangsu China; ^3^ Department of Endocrinology, Jiangsu Province Hospital of Chinese Medicine Affiliated Hospital of Nanjing University of Chinese Medicine Nanjing Jiangsu China

**Keywords:** bioinformatics, ferroptosis, immunity, osteoarthritis, quercetin, type 2 diabetes mellitus

## Abstract

This study investigated the underlying comorbidity mechanism between type 2 diabetes mellitus (T2DM) and osteoarthritis (OA), while also assessing the therapeutic potential of quercetin for early intervention and treatment of these two diseases. The shared genes were obtained through GEO2R, limma and weighted gene co‐expression network analysis (WGCNA), and validated using clinical databases and the area under the curves (ROC). Functional enrichment analysis was conducted to elucidate the underlying mechanisms of comorbidity between T2DM and OA. The infiltration of immune cells was analysed using the CIBERSORT algorithm in conjunction with ESTIMATE algorithm. Subsequently, transcriptional regulation analysis, potential chemical prediction, gene‐disease association, relationships between the shared genes and ferroptosis as well as immunity‐related genes were investigated along with molecular docking. We identified the 12 shared genes (EPHA3, RASIP1, PENK, LRRC17, CEBPB, EFEMP2, UBAP1, PPP1R15A, SPEN, MAFF, GADD45B and KLF4) across the four datasets. Our predictions suggested that targeting these shared genes could potentially serve as therapeutic interventions for both T2DM and OA. Specifically, they are involved in key signalling pathways such as p53, IL‐17, NF‐kB and MAPK signalling pathways. Furthermore, the regulation of ferroptosis and immunity appears to be interconnected in both diseases. Notably, in this context quercetin emerges as a promising drug candidate for treating T2DM and OA by specifically targeting the shared genes. We conducted a bioinformatics analysis to identify potential therapeutic targets, mechanisms and drugs for T2DM and OA, thereby offering novel insights into molecular therapy for these two diseases.

## 1 INTRODUCTION

Type 2 diabetes mellitus (T2DM) is characterized by hyperglycaemia and abnormal insulin secretion.^1^ Osteoarthritis (OA) is a prevalent degenerative joint disease in clinical practice, presenting with joint pain, limited function and a certain disability rate.^2^ It has been observed that there exists a significant correlation between T2DM and OA, with the incidence of OA being notably higher in the T2DM group compared to the non‐T2DM group.^3^ Dubey^4^ discovered an upregulation of matrix metalloproteinase (MMP)‐1 expression in knee joint tissue of T2DM mice, leading to intensified collagen degradation and accelerated articular cartilage degeneration. Neumann^5^ found an accelerated trend of knee cartilage degeneration in patients with T2DM when compared to healthy individuals without diabetes mellitus. Research has demonstrated that hyperglycaemia and insulin resistance are pivotal factors inducing OA in individuals with T2DM.^6^

Comprehensive analysis based on transcriptome data from some databases has become a useful method to identify novel differentially expressed genes (DEGs) and reveal their biological functions in diseases.^7^ There have also been bioinformatics studies on T2DM or OA recently. For example, Cui^8^ identified DEGs related to autophagy in T2DM. Yang^9^ verified the hub genes of T2DM by single‐cell sequencing. Wang^10^ identified DEGs related to ferroptosis in T2DM based on machine learning. Meng^11^ adopted machine learning to identify DEGs related to autophagy and apoptosis in OA. Li^12^ identified immune‐related genes of OA complicated with metabolic syndrome by bioinformatics analysis. Chu^13^ confirmed DEGs of central methylation in OA based on transcriptome data. However, the genetic mechanism between T2DM and OA is still unclear. Therefore, it is particularly important to identify new therapeutic targets for early diagnosis and specific intervention of T2DM and OA progression.

**FIGURE 1:**
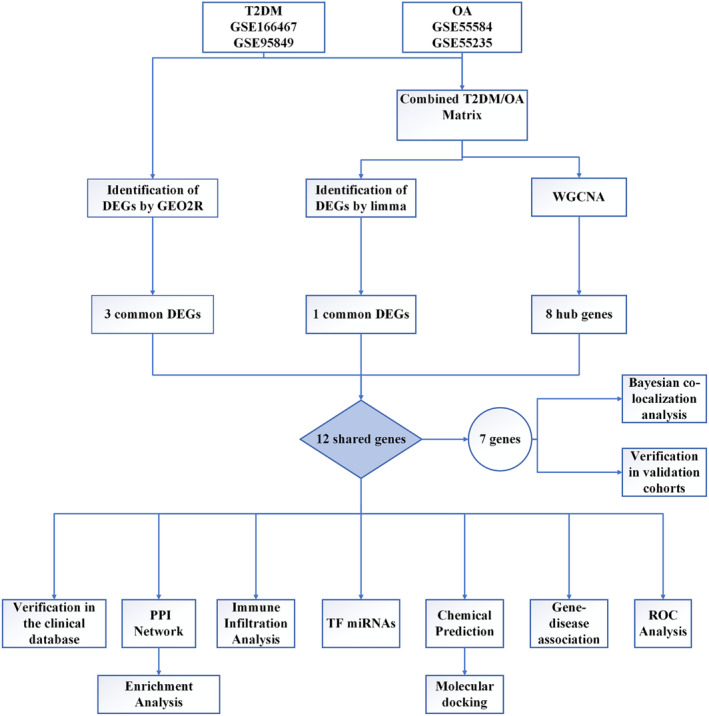
The protocol of our study procedure.

In recent years, machine learning methods have been extensively applied across various domains of bioinformatics. For instance, Wang^14^ developed a novel deep learning prediction model called DMFGAM by integrating the characteristics of molecular fingerprints and molecular graphs to predict Human ether‐a‐go‐go‐related gene (HERG) blockers. In 2022, Wang^15^ proposed GCNCRF, a method based on graph convolutional neural (GCN) network and conditional random field (CRF), for predicting lncRNA–miRNA interactions. Sun^16^ introduced GCNAT, a graph convolutional network with graph attention network (GCNAT), to forecast potential associations between disease‐related metabolites. This study aims to systematically analyse genes, miRNA, transcription factors (TFs), signal transduction pathways and related drugs using multiple bioinformatics analyses in order to elucidate the underlying mechanism of comorbidity between T2DM and OA. Notably different from previous studies that focused on screening DEGs in either T2DM or OA diseases through bioinformatics analysis are three aspects: Firstly, the disease‐related phenotypes are different from before. Secondly, distinct criteria were employed for DEGs selection. Last but not least, this study combined T2DM and OA, which revealed the comorbidity mechanism of T2DM and OA for the first time. The findings from this study provide valuable theoretical insights for clinical research on T2DM and OA. The study protocol is illustrated in Figure 1.

## 2 METHOD

### 2.1 Bioinformatics analysis

#### 2.1.1 Data processing and differential expression analysis

In this study, we downloaded the gene expression data of T2DM and OA from the GEO database (https://www.ncbi.nlm.nih.gov/geo/). As the training cohort, we selected GSE166467 and GSE95849, which comprised expression data from 32 patients with T2DM and 38 healthy tissues for studying T2DM. For investigating OA, we chose GSE55584 and GSE55235, encompassing expression data from 29 patients with OA and 23 healthy tissues. As validation cohorts, we obtained GSE29221 and GSE12021 from the GPL6947 and GPL96 platforms, respectively. The characteristics of the datasets used in this study are presented in Table 1. The differential expression analysis between T2DM and OA data set was analysed by GEO2R (https://www.ncbi.nlm.nih.gov/geo/geo2r/), and the difference multiple was set as 1.5, *p* < 0.05 as the screening conditions of differential expression genes (DEGs). The common DEGs of four data sets were obtained by Venn diagram (https://sourceforge.net/projects/venn/). After applying the sva R package to remove batch effects between T2DM and OA datasets, we utilized the limma R package for differential expression analysis of genes in the combined matrix, employing |log2(FC)| > 1 and *p* < 0.05 as screening criteria. The DEGs were visualized using the pheatmap R package for constructing a heatmap, while the ggplot2 R package was employed to generate a volcano plot of DEGs.

**TABLE 1:** Characteristics of the datasets used in this study.

Dataset	Database	Platform	Sample
GSE166467	GEO	GPL10558	26 patients with T2DM and 26 healthy tissues
GSE95849	GEO	GPL22448	6 patients with T2DM and 12 healthy tissues
GSE29221	GEO	GPL6947	12 patients with T2DM and 12 healthy tissues
GSE55584	GEO	GPL96	9 patients with OA and 13 healthy tissues
GSE55235	GEO	GPL96	20 patients with OA and 10 healthy tissues
GSE12021	GEO	GPL96	44 patients with OA and 13 healthy tissues

#### 2.1.2 Weighted gene co‐expression network analysis (WGCNA)

WGCNA is an algorithm that can find co‐expressed gene modules with high biological significance and explore the relationship between gene networks and diseases.^17^ We used the goodSamplesGenes method of the WGCNA R package to remove outliers and samples, and further used WGCNA to build a scale‐free co‐expression network. Firstly, the sample clustering tree algorithm was utilized to filter out outliers and ensure the stability of co‐expression network construction. Additionally, the connections between genes in the co‐expression network followed a scale‐free distribution and then the optimal soft threshold *β* was determined using the Pick Soft Threshold function.

Secondly, the adjacency matrix was transformed into a topological overlapping matrix (TOM) in order to mitigate spurious correlations and noise. Subsequently, 1‐TOM was computed as a crucial biological indicator of co‐expression network interconnectivity and gene clustering distance. Thirdly, the dynamic tree‐cutting algorithm was employed to identify gene modules, each comprising a minimum of 20 genes. Finally, module characteristic values (ME) were calculated and hierarchically clustered. Similar modules were merged using a shear height threshold of 0.25 and distinguished by different colours. Specific modules were determined based on the module characteristic relationship (Pearson correlation between ME and clinical features). A characteristic heatmap depicting the clustering module was generated while calculating MM (Module Membership) values (correlation coefficient between gene significance and module significance) as well as GS (Gene Significance) values (correlation coefficient between gene expression levels and clinical characteristics).

#### 2.1.3 Construction of PPI network and enrichment analysis

In this study, we defined the DEGs and hub genes as the shared genes. To further investigate the interrelationships among these shared genes, a protein–protein interaction (PPI) network was constructed using the GeneMANIA (http://genemania.org/).^18^

To identify potential functions among the shared genes of T2DM and OA, we utilized the GO annotation of genes in the R package org.Hs.eg.db as the background set. The genes were then mapped to this background set, and gene set enrichment analysis was performed using the clusterProfiler R package. A minimum gene set size of 5 and a maximum gene set size of 5000 were applied, with statistical significance defined as *p* < 0.05 and FDR < 0.1. The results were visualized using the ggplot2 R package. Additionally, we conducted single‐gene GSEA to investigate possible immune‐related mechanisms associated with these shared genes by employing GSEA software obtained from the GSEA website (http://software.broadinstitute.org/gsea/index.jsp). Samples were categorized into high expression groups and low expression groups based on expression levels of these shared genes. Based on the gene expression profile and phenotypic grouping, the minimum gene set was set to 5, while the maximum gene set was set to 5000, with 1000 resamples, *p* < 0.05 and FDR < 0.25 were considered statistically significant.

#### 2.1.4 Verification of the shared genes in the clinical database

In order to further investigate the reliability of the shared genes, we employed boxplots to validate their expression in the combined matrix of OA and T2DM. The shared genes were subjected to verification using the Nephroseq database (https://www.nephroseq.org//). A comparison between healthy individuals and those with DM was conducted through an unpaired *t*‐test to assess the expression levels of these shared genes. Additionally, Pearson correlation analysis was utilized to explore any potential associations between the shared genes and fasting blood glucose levels in patients with DM.

#### 2.1.5 Immune infiltration analysis

The CIBERSORT algorithm was employed to estimate the abundance of 22 infiltrating immune cell types in the combined T2DM and OA datasets, respectively. CIBERSORT is a deconvolution analysis method based on linear support vector regression principles that enable the prediction of cell type proportions from gene expression profiles. A significance level of *p* < 0.05 was adopted, and the results were visualized using the barplot R package. Additionally, the ESTIMATE algorithm was utilized to calculate immune scores and estimate scores for both T2DM and OA datasets.

#### 2.1.6 Transcription regulation analysis and potential chemical prediction

The NetworkAnalyst platform is an online tool for visual analysis of gene expression profiles, facilitating the exploration of transcription factors (TFs) that regulate gene transcription and miRNAs that control post‐transcriptional gene expression. Their activities are very important for gaining molecular insight.^19^ We collected the interaction network between TFs and the shared genes from the JASPAR database on the network analysis platform, and the data of miRNA‐shared genes interaction from TarBase.^20^ Shared gene‐chemical interaction was obtained by the CTD database.

#### 2.1.7 Gene‐disease association

Gene‐disease associations suggest that certain diseases may share common molecular mechanisms in their progression. GenDoma, the first comprehensive database of human genes and diseases in China, integrates over 70 databases and text information mined by self‐built models to analyse human genes, disease molecular mechanisms, transformations and applications from multiple dimensions to fully support precision medicine. In this study, we retrieved the shared gene‐disease associations from the Gendoma website (https://ai.citexs.com).

#### 2.1.8 Relationship between the shared genes and ferroptosis, immunity‐related genes

In order to elucidate the potential mechanism underlying the interplay between ferroptosis, immunity and the two diseases, we conducted an analysis on the correlation between the 12 shared genes and the top 20 genes associated with ferroptosis and immunity. The relevant genes related to ferroptosis and immunity were sourced from the GeneCards database (https://www.genecards.org/) (Table S1).

#### 2.1.9 ROC analysis

The ROC curves for the shared genes in both diseases were generated using the ROC package and corresponding datasets. The AUC was then calculated to assess its diagnostic efficacy in these two diseases, with an AUC value exceeding 0.5 considered indicative of a statistically significant difference.

#### 2.1.10 Molecular docking

In order to validate the interaction between the predicted hub chemical quercetin and the shared genes, we obtained the ligand's structural files of quercetin in mol2 format from the PubChem database (https://pubchem.ncbi.nlm.nih.gov/), and downloaded the 3D structural files of the shared genes (receptors) in pdb format from the PDB database (https://www.rcsb.org/).

The receptors were dehydrated, hydrogenated and charged using AutoDockTools software. Subsequently, they were exported to a pdbqt format file, while the ligand was also output in pdbqt format. Next, we imported the processed files into AutoDockTools software and determined the docking box (grid box parameters can be found in Table S2). Finally, we calculated the binding energy. The results of docking binding energy were visualized in the form of heatmap. The ligand‐receptor structure in the top 4 of binding energy were visualized by PyMOL software.

#### 2.1.11 Statistical analysis

Statistical analysis was performed using R (4.1.2) and GraphPad Prism (8.0.1). One‐way ANOVA was utilized to compare groups, while independent sample *t*‐tests or Mann–Whitney *U* tests were employed to assess systematic differences in continuous variables. *p* < 0.05 is statistically significant.

### 2.2 Bayesian co‐localization analysis

Bayesian co‐localization analysis is a method to evaluate whether there is common causal variation in two phenotypic regions. We used the ‘coloc’ R package^21^ to co‐locate the seven genes (EPHA3, CEBPB, UBAP1, PPP1R15A, MAFF, GADD45B and KLF4) which were reported to be related to the pathological mechanism of T2DM and OA, and the co‐location analysis provides four hypothetical posterior probabilities of whether a single variable is shared by two traits. The criterion for determining gene co‐location is to assume that 4 (PPH4) score is greater than 0.8.^22^ The GWAS data of OA (ebi‐a‐GCST90038686) and T2DM (ebi‐a‐GCST006867) came from the IEU OpenGWAS (https://gwas.mrcieu.ac.uk/).

## 3 RESULTS

### 3.1 Bioinformatics analysis

#### 3.1.1 Differential expression analysis

**FIGURE 2:**
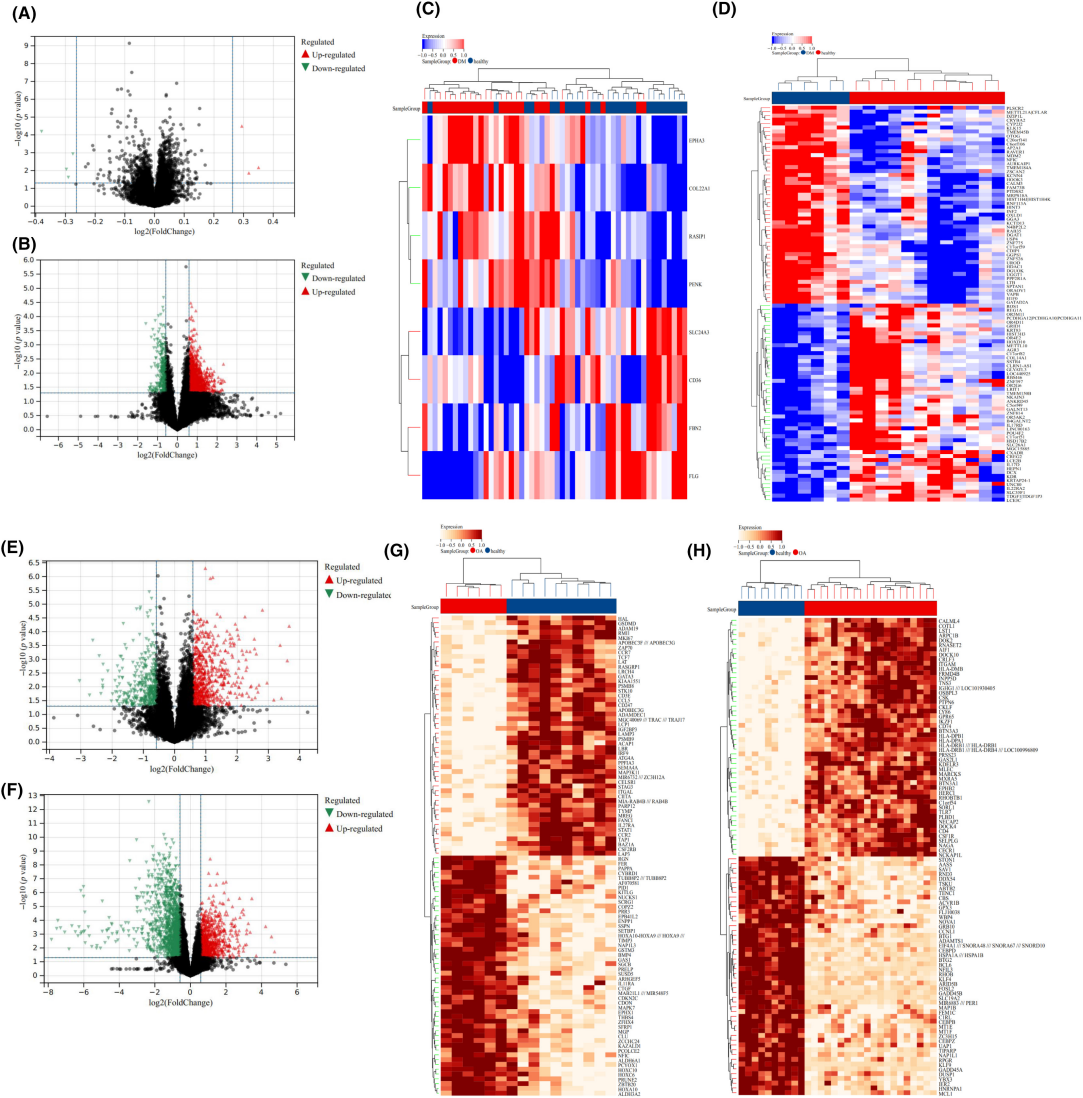
Identification of DEGs in the four data sets (T2DM and OA). (A) Volcanic map of DEGs in GSE166467 (T2DM). (B) Volcanic map of DEGs in GSE95849 (T2DM). (C) Heatmap of DEGs in GSE166467 (T2DM). Red represents the DM group, brown represents the healthy group, and the redder the colour, the higher the gene expression in the sample. (D) Heatmap of DEGs in GSE95849 (T2DM). Red represents the healthy group, brown represents the DM group, and the redder the colour, the higher the gene expression in the sample. (E) Volcanic map of DEGs in GSE55584 (OA). (F) Volcanic map of DEGs in GSE55235 (OA). (G) Heatmap of DEGs in GSE55584 (OA). Red represents the OA group, brown represents the healthy group, and the oranger the colour, the higher the gene expression in the sample. (H) Heatmap of DEGs in GSE55235 (OA). Red represents the OA group, brown represents the healthy group, and the oranger the colour, the higher the gene expression in the sample. In the ABEF diagram, the red triangle in the diagram represents the upregulated genes and the green triangle represents the downregulated genes.

**FIGURE 3:**
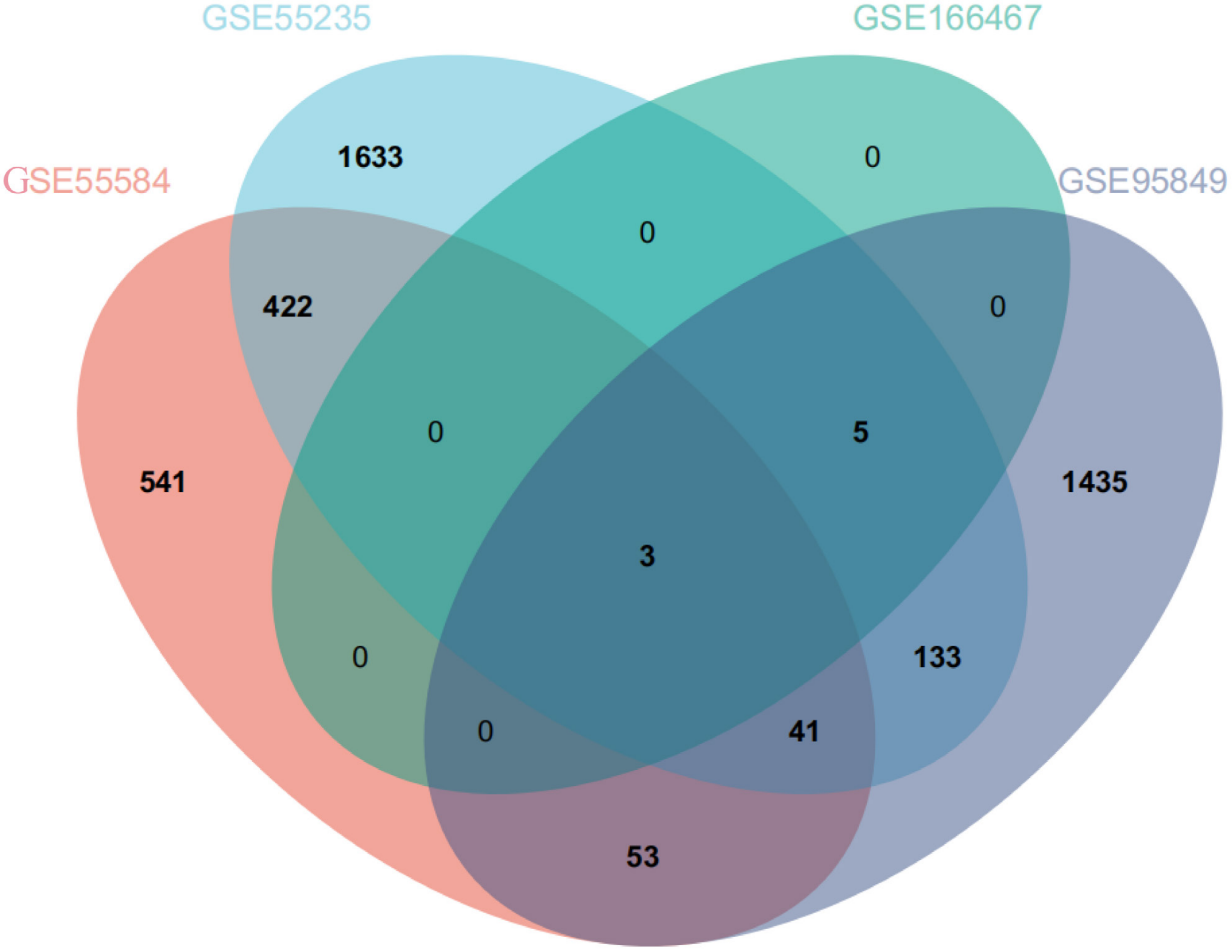
Venn diagram of DEGs between the four data sets (T2DM and OA). Pink represents the GSE55584 dataset, blue represents the GSE55235 dataset, green represents the GSE166467 dataset, and purple represents the GSE95849 dataset.

By using GEO2R, a total of 1678 DEGs were screened out in T2DM, including 1361 upregulated genes and 314 downregulated genes, while a total of 3299 DEGs were screened out in OA, including 1511 upregulated genes and 1788 downregulated genes. The volcanic map and heatmap of DEGs in the four data sets are shown in Figure 2A–H, respectively. Venn diagram was used to screen out three common DEGs both in T2DM and OA (Figure 3).

**FIGURE 4:**
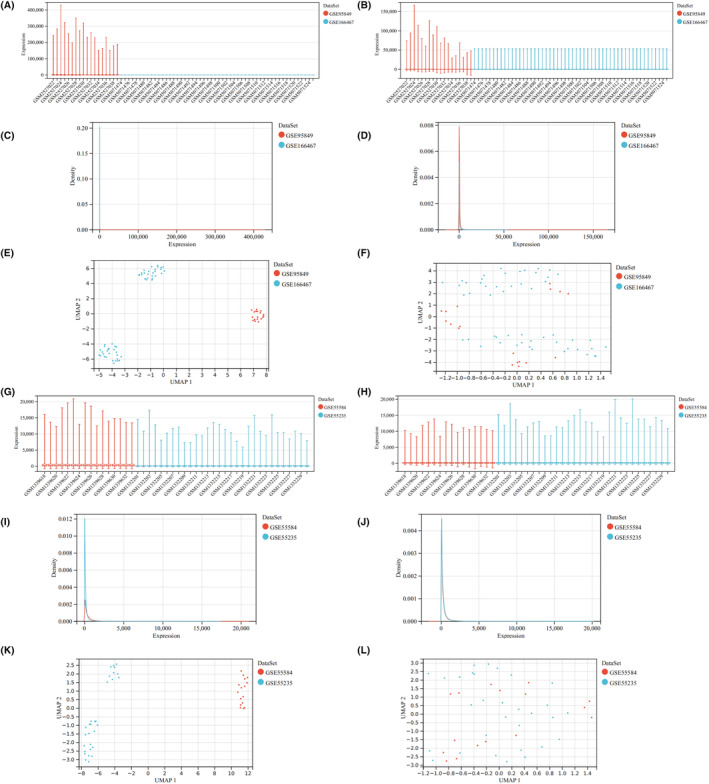
Analysis of two T2DM/OA data sets before and after batch effect elimination. (A) Boxplots of two T2DM datasets prior to batch effect removal. (B) Boxplot of two T2DM datasets after batch effect removal. (C) Density plot of two T2DM datasets before batch effect removal. (D) Density plot of two T2DM datasets after batch effect removal. (E) UMAP plot of two T2DM datasets before batch effect removal. (F) UMAP plot of two T2DM datasets after batch effect removal. (G) Boxplot of two OA datasets before batch effect removal. (H) Boxplot of two OA datasets after batch effect removal. (I) Density plot of two OA datasets before batch effect removal. (J) Density plot of two OA datasets after batch effect elimination. (K) UMAP plot of two OA datasets before batch effect removal. (L) UMAP plot of two OA datasets after batch effect removal.

**FIGURE 5:**
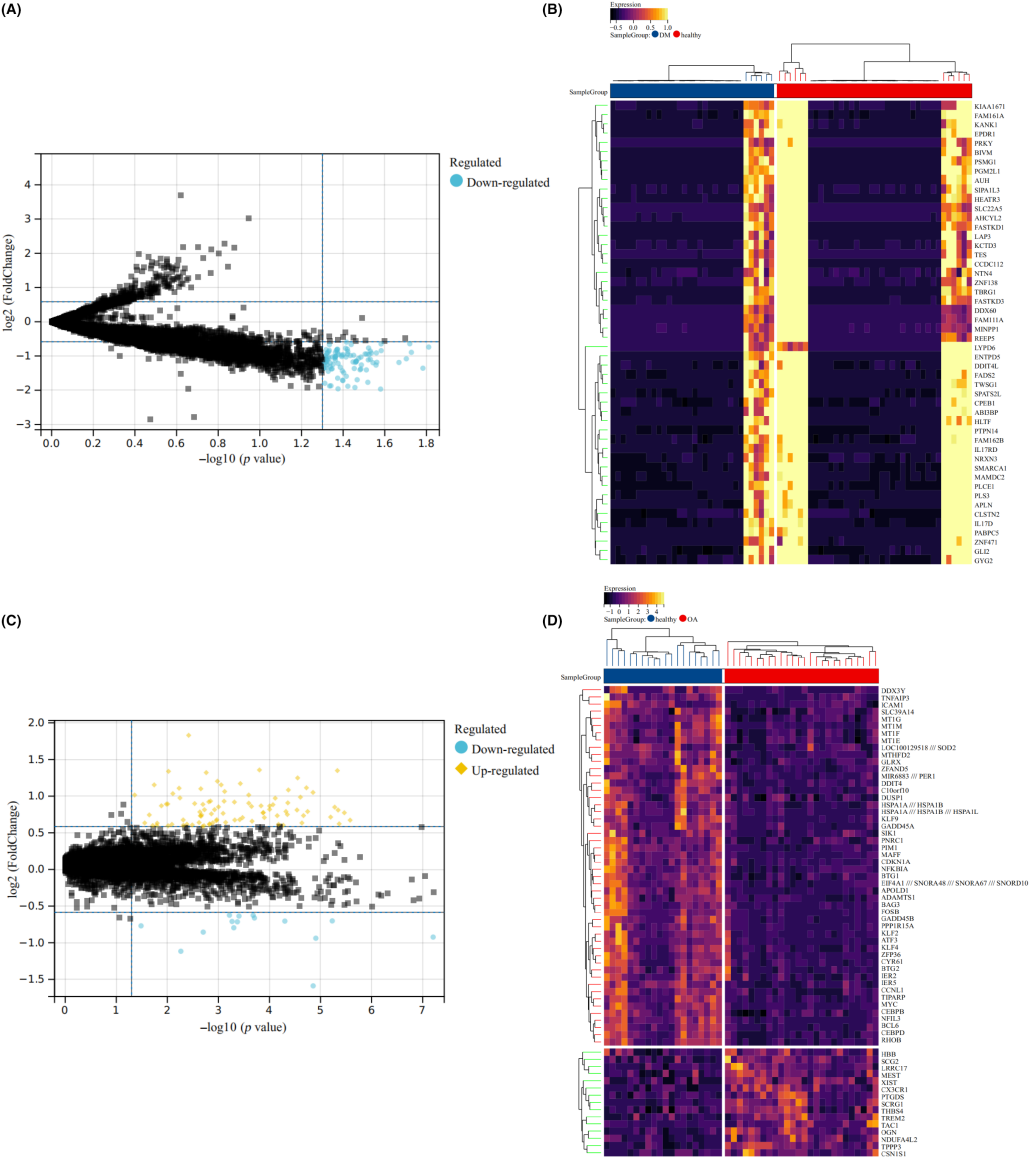
Identification of DEGs from the combined T2DM and OA data sets. (A) Volcanic map of DEGs in the combined T2DM data set. The blue circle represents downregulated genes. (B) Heatmap of DEGs in the combined T2DM data set. Red represents the healthy group, brown represents the DM group, and the yellower the colour, the higher the gene expression in the sample. (C) Volcanic map of DEGs in the combined OA data set. The blue circle represents downregulated genes, and the orange diamond represents upregulated genes. (D) Heatmap of DEGs in the combined OA data set. Red represents the OA group, brown represents the healthy group, and the yellower the colour, the higher the gene expression in the sample.

**FIGURE 6:**
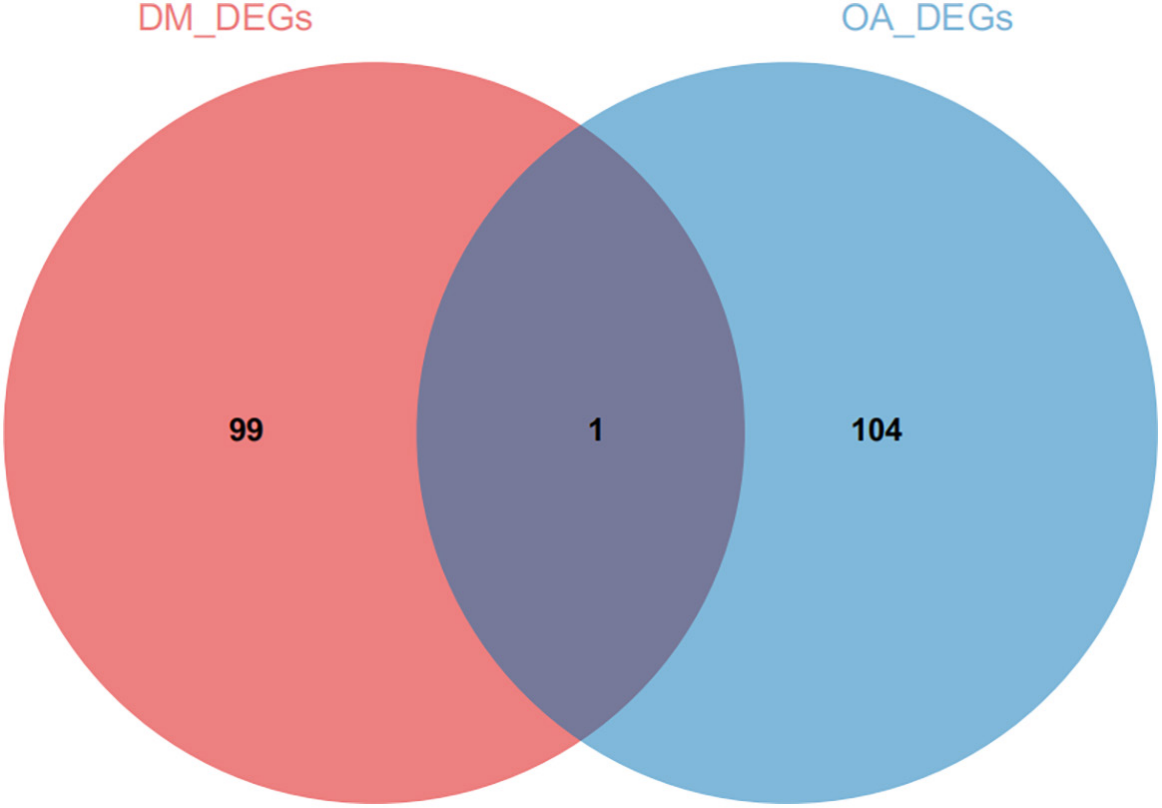
Identification of common DEGs from the combined T2DM and OA data sets. Pink represents DEGs in the combined T2DM dataset, and blue represents DEGs in the combined OA dataset.

Furthermore, we integrated two T2DM/OA datasets to investigate the DEGs. By comparing the boxplot, density diagram and UAMP diagram before and after batch effect removal, it was observed that the sample distribution of each dataset exhibited significant dissimilarity prior to batch effect correction. However, post‐correction, the data distributions of both datasets tended to converge towards consistency (Figure 4A–L). We identified a total of 100 DEGs from the combined T2DM matrix and 105 DEGs from the combined OA matrix (Figure 5A–D). Notably, one common DEG was found in both the combined T2DM and OA matrices (Figure 6).

#### 3.1.2 WGCNA

**FIGURE 7:**
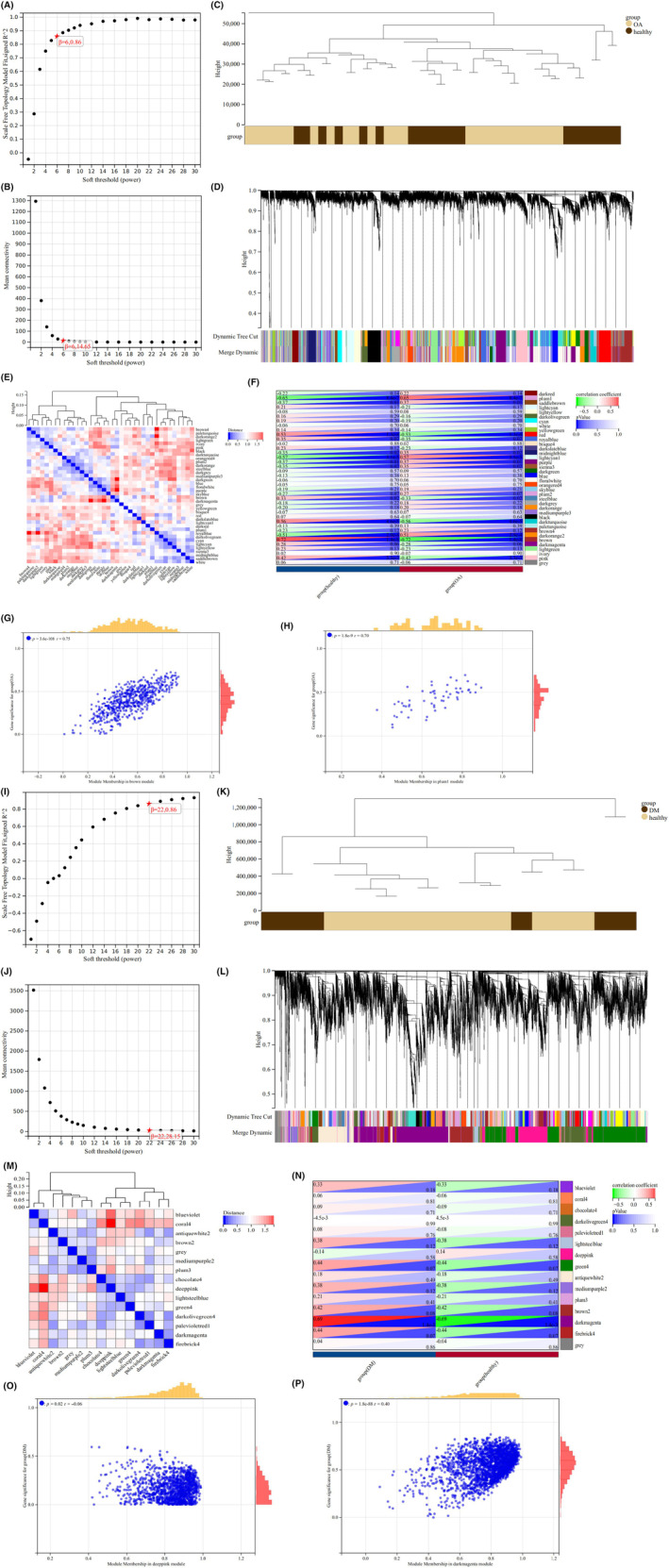
Results of WGCNA. (A) Scale independence of different soft thresholds in the combined OA dataset. *β* = 6, 0.86 means when soft‐threshold powers (*β*) = 6, the scale‐free index is 0.86. When *β* = 6, the co‐expression network is a scale‐free network. (B) Average connectivity of different soft thresholds in the combined OA dataset. *β* = 6, 14.65 means when soft‐threshold powers (*β*) = 6, the mean connectivity is 14.65. (C) Cluster diagram of grouped samples in the combined OA dataset. Brown represents the OA group, and dark brown represents the healthy group. (D) Dendrogram of genes clustering in the combined OA dataset. Dendrogram of genes clustered based on the measurement of dissimilarity (1‐TOM). The colour band shows the results obtained from the automatic single‐block analysis. (E) Heatmap of modular feature vector clustering in the combined OA dataset. The redder the colour, the longer the distance between modules. (F) Correlation heatmap between module and phenotype in the combined OA dataset. The colour from deep green to deep red refers to the correlation coefficient from small to large and the colour from deep blue to white refers to the *p*‐value. (G) Scatter diagram of the correlation between GS and MM of the brown module in the combined OA dataset. The horizontal axis represents Module Membership, and the vertical axis represents Gene Significance. (H) Scatter diagram of the correlation between GS and MM of the plum1 module in the combined OA dataset. The horizontal axis represents Module Membership, and the vertical axis represents Gene Significance. (I) Scale independence of different soft thresholds in the combined T2DM dataset. *β* = 22, 0.86 means when soft‐threshold powers (*β*) = 22, the scale‐free index is 0.86. When *β* = 22, the co‐expression network is a scale‐free network. (J) Average connectivity of different soft thresholds in the combined T2DM dataset. When *β* = 22, the co‐expression network is a scale‐free network. (K) Cluster diagram of grouped samples in the combined T2DM dataset. Brown represents the healthy group, and dark brown represents the DM group. (L) Tree diagram of gene clustering in the combined T2DM dataset. Dendrogram of genes clustered based on the measurement of dissimilarity (1‐TOM). The colour band shows the results obtained from the automatic single‐block analysis. (M) Heatmap of modular feature vector clustering in the combined T2DM dataset. The redder the colour, the longer the distance between modules. (N) Correlation heatmap between module and phenotype in the combined T2DM dataset. The colour from deep green to deep red refers to the correlation coefficient from small to large and the colour from deep blue to white refers to the *p*‐value. (O) Scatter diagram of the correlation between GS and MM of the deep pink in the combined T2DM dataset. The horizontal axis represents Module Membership, and the vertical axis represents Gene Significance. (P) Scatter diagram of the correlation between GS and MM of the darkmagenta in the combined T2DM dataset. The horizontal axis represents Module Membership, and the vertical axis represents Gene Significance.

In the combined OA matrix, soft threshold *β* = 6 was selected (Figure 7A–C), and 38 gene modules were identified by dynamic tree cutting (Figure 7D,E). The correlation between the gene module and clinical feature showed that the plum1 module had the highest positive correlation with OA (*R* = 0.65, *p* = 8.4e‐7), while the brown module had the highest negative correlation with OA (*R* = −0.72, *p* = 1.4e‐8) (Figure 7F). The scatter plot of the correlation between MM and GS showed that the genes were highly correlated with the two modular phenotypes (Figure 7G,H).

**FIGURE 8:**
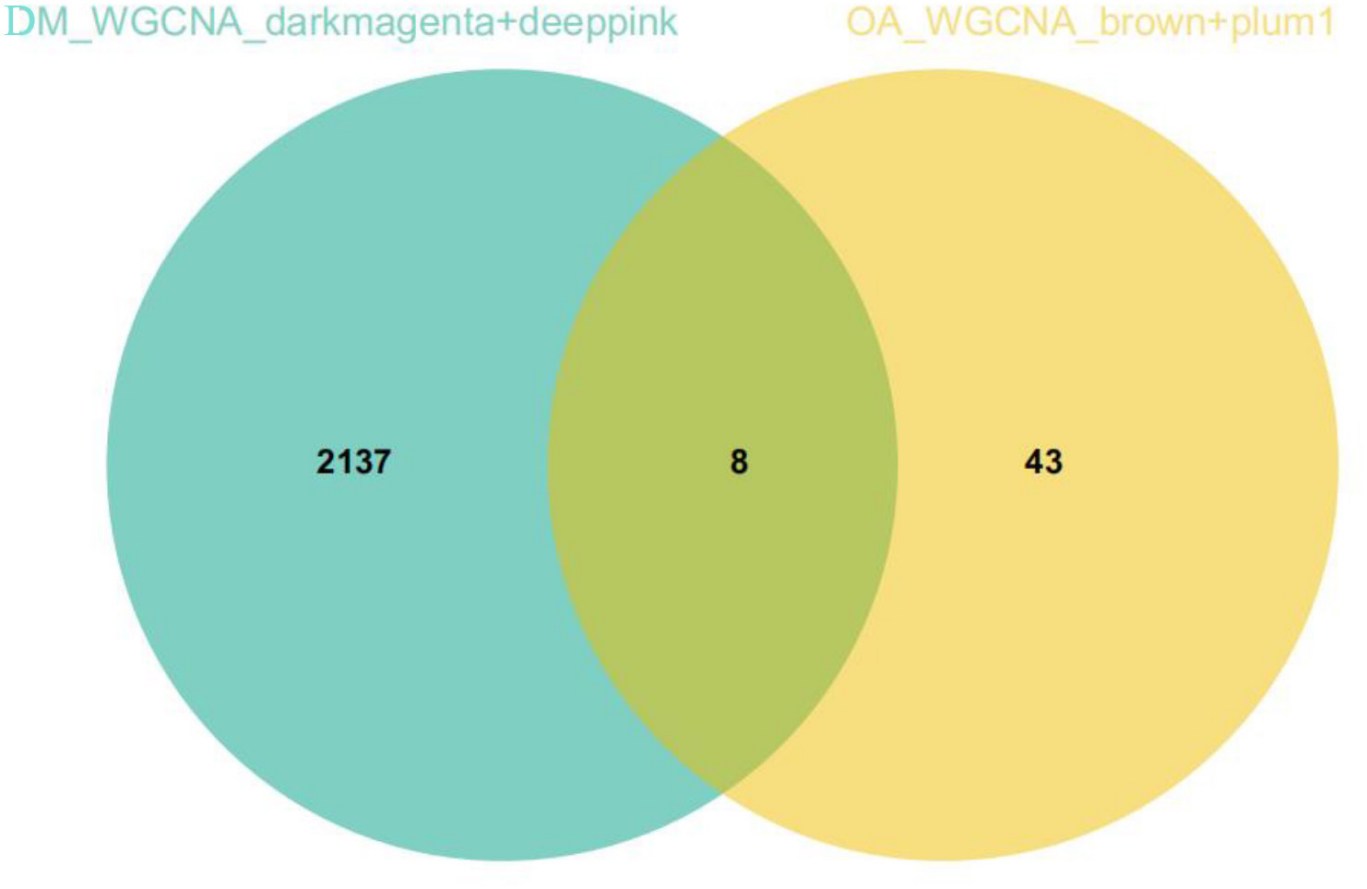
Venn diagram of hub genes by WGCNA. Green represents genes in darkmagenta and deep‐pink modules, and yellow represents genes in plum1 and brown modules.

In the combined T2DM matrix, the soft threshold *β* = 22 was selected (Figure 7I–K), and 15 gene modules were identified by dynamic tree‐cutting (Figure 7L,M). The correlation between the gene module and clinical feature showed that the darkmagenta module had the highest positive correlation with DM (*R* = 0.69, *p* = 1.4e‐3), while the deep‐pink module had the highest negative correlation with DM (*R* = −0.14, *p* = 0.58) (Figure 7N). Similarly, the scatter plot of the correlation between MM and GS showed that the genes were highly correlated with the two modular phenotypes (Figure 7O,P). Therefore, we selected the above four modules as the key modules to identify the hub genes. There were eight genes both in the T2DM matrix and OA matrix that were identified as hub genes from the four modules (Figure 8).

#### 3.1.3 Construction of PPI network and enrichment analysis

**FIGURE 9:**
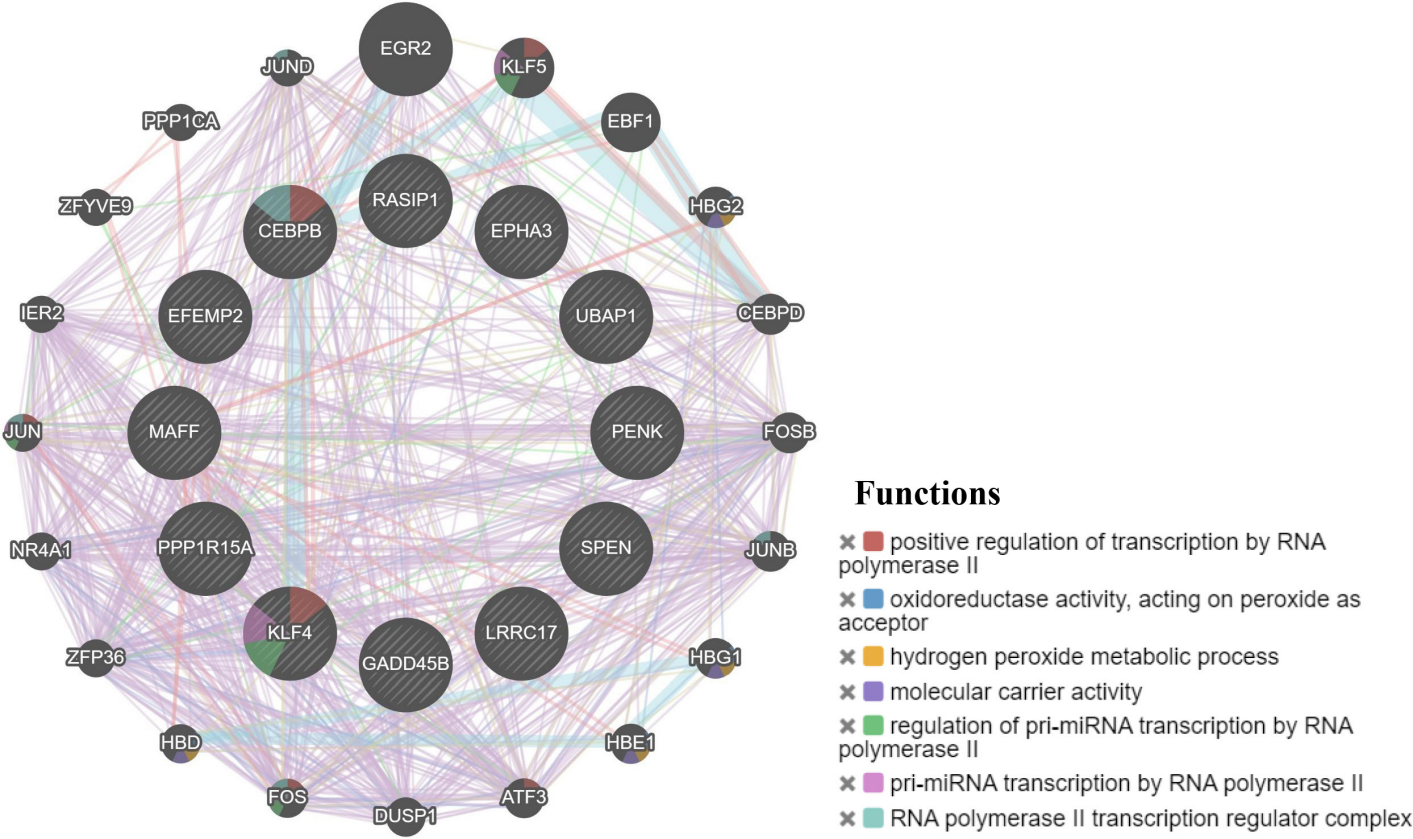
Construction of PPI network. Different colours in the gene circle represent different functions of genes.

By constructing the PPI network, we found that KLF4 and CEBPB were in the hub position. The shared genes could be related to each other through molecular carrier activity, hydrogen peroxide metabolic process and positive regulation of transcription by RNA polymerase II (Figure 9).

**FIGURE 10:**
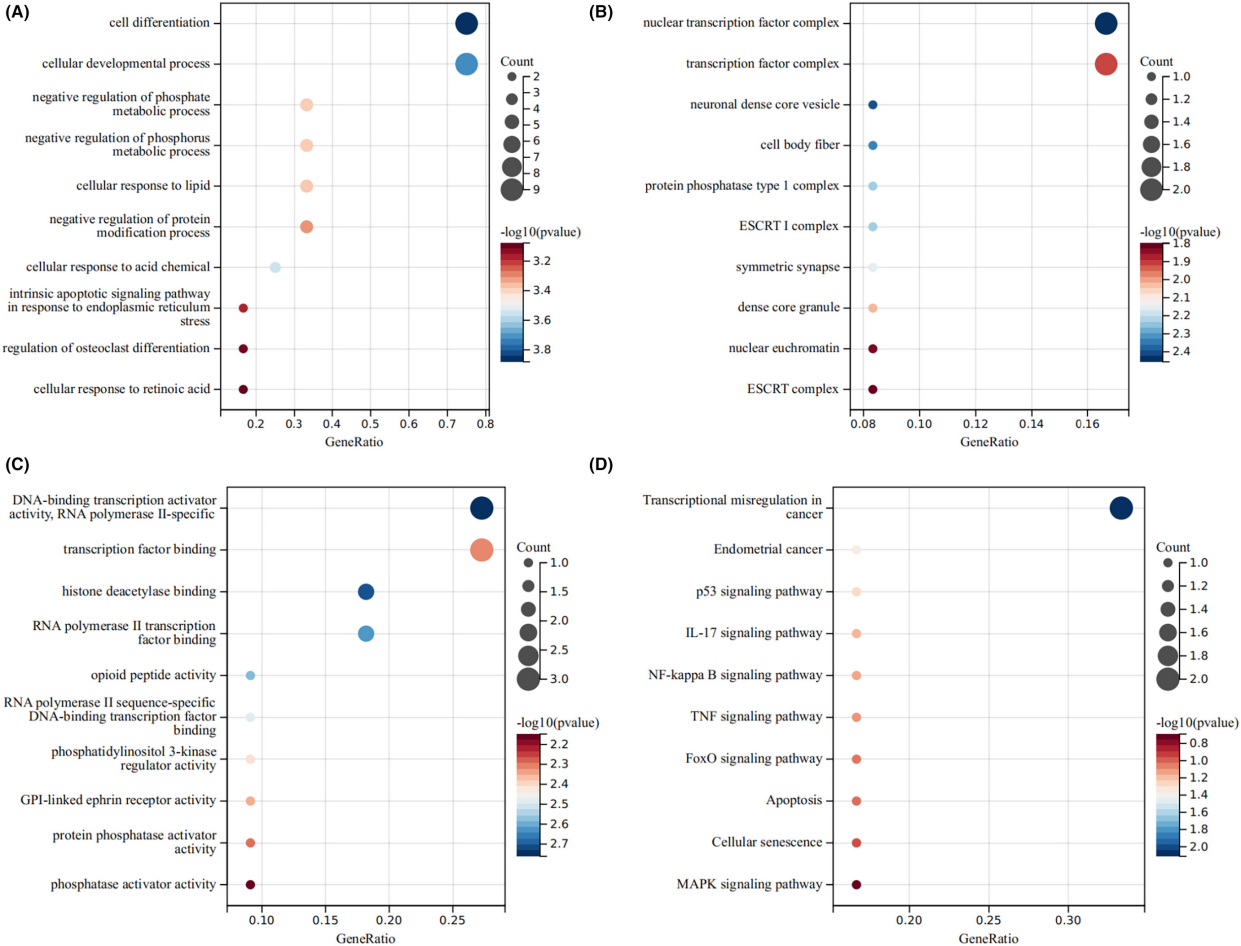
Enrichment analysis of the shared genes. (A) represents the biological process bubble diagram of the shared genes. (B) represents the cellular component bubble diagram of the shared genes. (C) represents the molecular function bubble diagram of the shared genes. (D) represents the KEGG bubble diagram of the shared genes. The bubble size represents the number of hub genes enrichment, and the colour depth represents the level of significance.

Functional enrichment analysis showed that the biological process of the shared genes was mainly enriched in cell differentiation, cellular development process and cellular response to lipid (Figure 10A). Cellular component were mainly enriched in cell body fibre, transcription factor complex and nuclear transcription factor complex (Figure 10B). Molecular function were mainly enriched in transcription factor binding and opioid peptide activity (Figure 10C). KEGG analysis is involved in the p53, IL‐17, NF‐kB and MAPK signalling pathway (Figure 10D).

**FIGURE 11:**
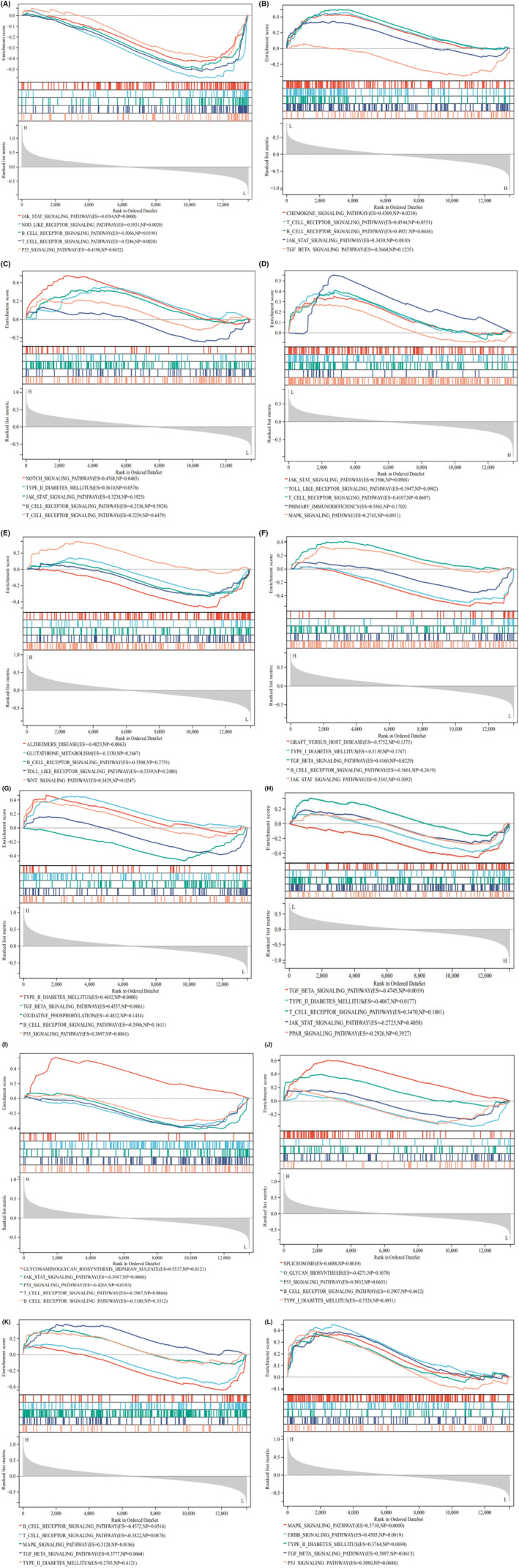
GSEA between the shared genes high and low subtypes. (A) GSEA of EPHA3 expression level. (B) GSEA of RASIP1 expression level. (C) GSEA of PENK expression level. (D) GSEA of LRRC17 expression level. (E) GSEA of CEBPB expression level. (F) GSEA of EFEMP2 expression level. (G) GSEA of UBAP1 expression level. (H) GSEA of PPP1R15A expression level. (I) GSEA of SPEN expression level. (J) GSEA of MAFF expression level. (K) GSEA of GADD45B expression level. (L) GSEA of KLF4 expression level. The leftmost represents the shared genes high subtypes, the rightmost represents the shared genes‐low subtypes.

GSEA analysis determines the underlying signal immune‐related pathway between the shared genes high and low subtypes, and the results showed that the EPHA3, PENK, CEBPB, EFEMP2, UBAP1, SPEN, MAFF, GADD45B and KLF4 high expression group were highly enriched in B‐cell receptor signalling pathway, T‐cell receptor signalling pathway and JAK–STAT signalling pathway. The high expression groups of PENK, EFEMP2, UBAP1, MAFF and KLF4 were mainly concentrated on diabetes mellitus (DM). The PPP1R15A low expression group was highly enriched in T2DM (Figure 11A–L). The aforementioned findings suggested a close association between the shared genes high subtype and both DM and immune infiltration, thereby raising the possibility that modulation of the immune microenvironment by these shared genes may hold therapeutic potential for T2DM and OA.

#### 3.1.4 Verification of the shared genes in the clinical database

**FIGURE 12:**
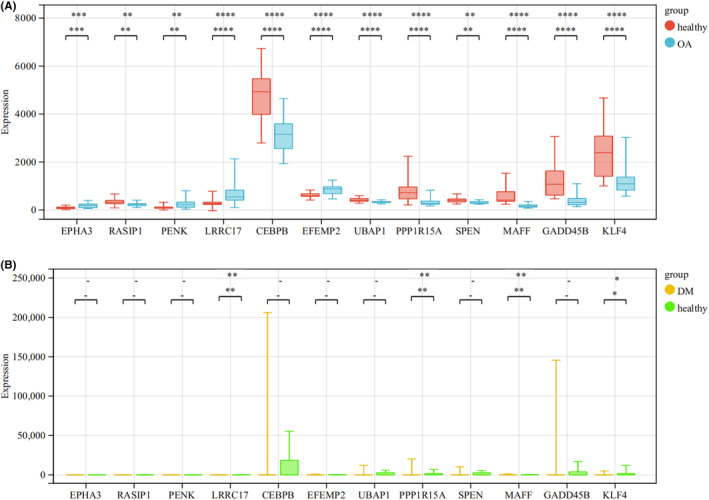
Verification of the shared genes in the combined T2DM and OA data sets. (A) Expression of the shared genes between the healthy and OA groups in the combined OA data set. Red represents the healthy group, blue represents the OA group. (B) Expression of the shared genes between the healthy and DM groups in the combined T2DM data set. Yellow represents the DM group, and green represents the healthy group. *p < 0.05, **p < 0.01, ***p < 0.001, ****p < 0.0001, −, no significant difference.

**FIGURE 13:**
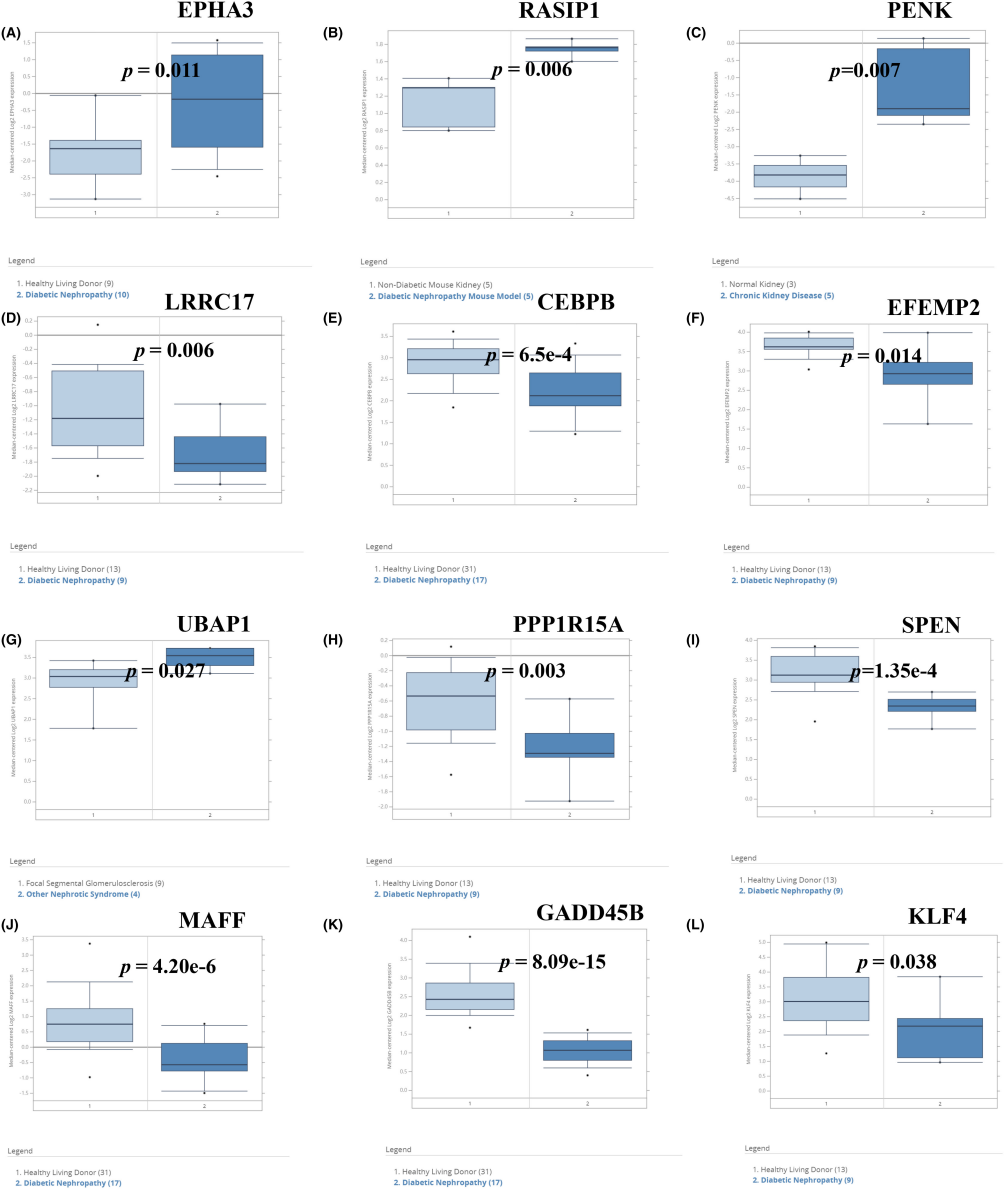
Results of the shared genes in the Nephroseq database. Dark blue represents chronic kidney disease, and light blue represents normal samples.

**FIGURE 14:**
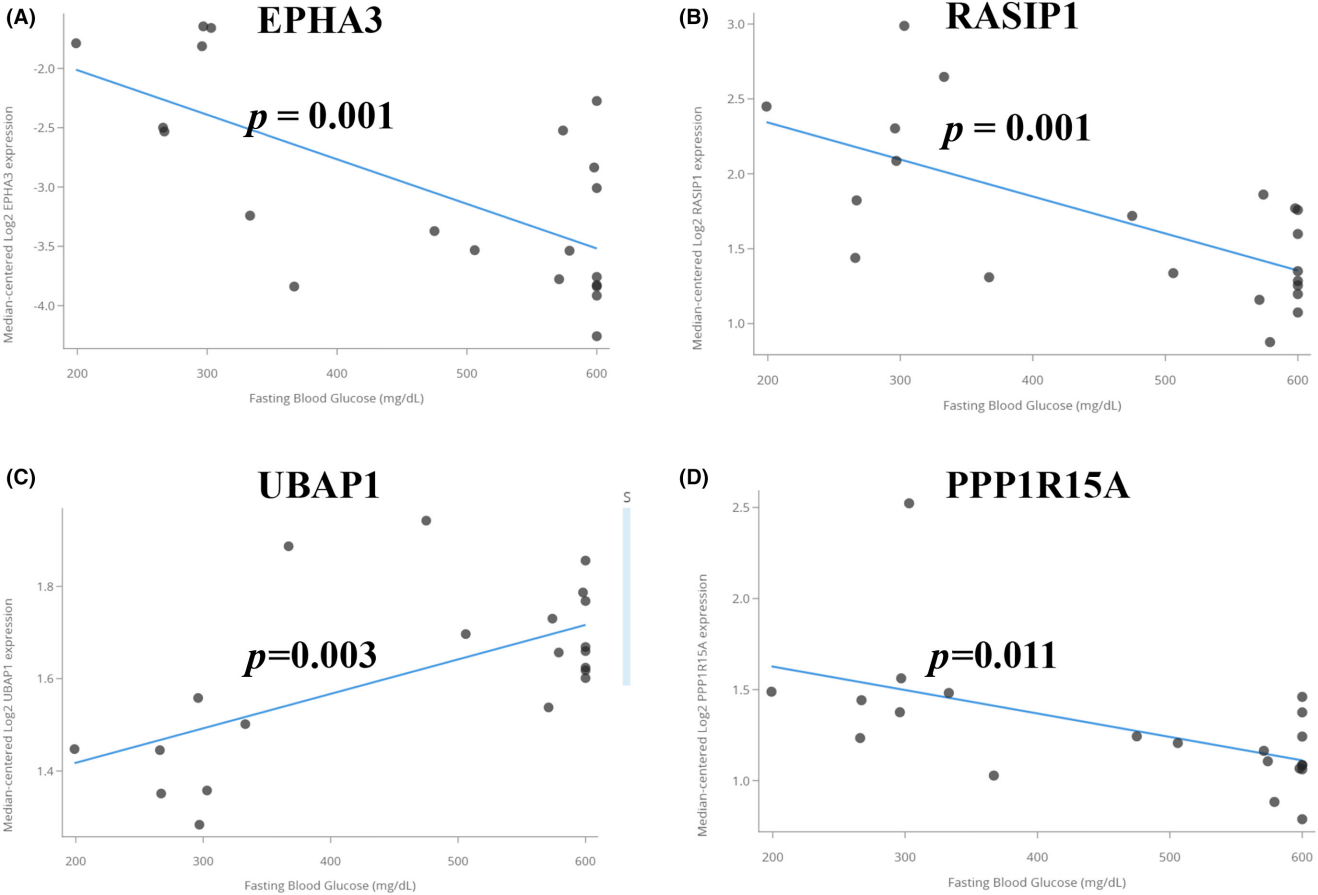
Relationship between the shared genes and fasting blood glucose in DM patients. The horizontal axis represents fasting blood glucose, and the vertical axis represents gene expression.

According to the boxplot, we found that EPHA3, PENK, LRRC17 and EFEMP2 were highly expressed in the OA group, while RASIP1, CEBPB, UBAP1, PPP1R15A, SPEN, MAFF, GADD45B and KLF4 were lowly expressed in the OA group, and the difference was statistically significant (*p* < 0.05) (Figure 12A). The expression of LRRC17, PPP1R15A, MAFF and KLF4 was higher in the DM group (*p* < 0.01), while KLF4 was lowly expressed in the DM group (*p* < 0.05) (Figure 12B). We verified the seven shared genes which were reported to be related to the pathological mechanism of T2DM and OA in the validation cohorts and found that CEBPB, MAFF and GADD45B were lowly expressed in the OA group (*p* < 0.05) (Figure S1A), while EPHA3, UBAP1 and KLF4 were lowly expressed in the DM group (*p* < 0.05) (Figure S1B). The correlation between the shared genes and diabetic nephropathy (DN) was verified by using the Nephroseq clinical database. The results showed that compared with normal kidney tissues, the mRNA expression of EPHA3, RASIP1, PENK and UBAP in DM patients were significantly upregulated (*p* < 0.05), while the mRNA expression of LRRC17, CEBPB, EFEMP2, PPP1R15A, SPEN, MAFF, GADD45B and KLF4 was significantly downregulated (*p* < 0.05) (Figure 13A–L). Pearson correlation analysis showed that the mRNA expression of UBAP1 was positively correlated with the fasting blood glucose level of DM patients, while the mRNA expression of EPHA3, RASIP1 and PPP1R15A were negatively correlated (Figure 14A–D). There was no data on the relationship between other shared genes and fasting blood glucose in the Nephroseq database.

#### 3.1.5 Immune infiltration analysis

**FIGURE 15:**
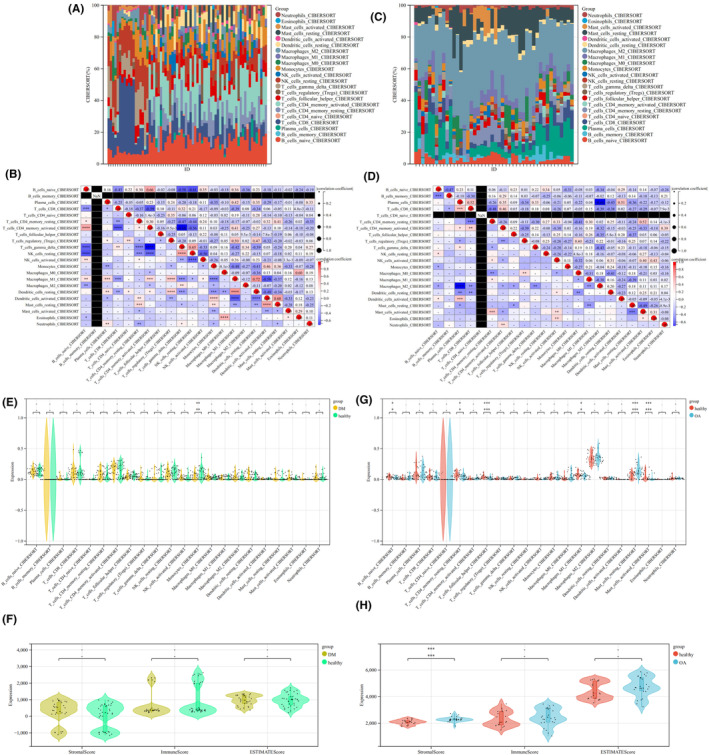
Immune infiltration analysis. (A) The proportion of 22 kinds of immune cells in different samples visualized from the barplot in the combined T2DM data set. (B) Heatmap of immune cell infiltration in the combined T2DM data set. The redder the colour, the stronger the correlation between the two immune cells, and the bluer the colour, the weaker the correlation. (C) The proportion of 22 kinds of immune cells in different samples visualized from the barplot in the combined OA data set. (D) Heatmap of immune cell infiltration in the combined OA data set. The redder the colour, the stronger the correlation between the two immune cells, and the bluer the colour, the weaker the correlation. (E) Difference of immune cell infiltration between T2DM and healthy samples in the combined T2DM data set. (F) Difference of estimate score between T2DM and healthy samples in the combined T2DM data set. (E, F) Yellow represents the DM group, and green represents the healthy group. (G) Difference of immune cell infiltration between OA and healthy samples in the combined OA data set. (H) Difference of estimate score between OA and healthy samples in the combined OA data set. (G, H) Blue represents the OA group, and red represents the healthy group. *p < 0.05, **p < 0.01, ***p < 0.001, ****p < 0.0001, −, no significant difference.

The relationship between the combined T2DM data set and 22 kinds of immune cell infiltration was conducted by box chart and heatmap (Figure 15A,B). We found that activated Mast cells had the strongest positive correlation with activated dendritic cells (*r* = 0.68, *p* < 0.0001), while activated CD4 memory T cells had the strongest negative correlation with gamma T cells (*r* = −0.68, *p* < 0.001). The results of immune cell infiltration analysis between the combined OA data set and 22 kinds of immune cell showed that activated Mast cells had the strongest positive correlation with resting CD4 memory T cells (*r* = 0.52, *p* < 0.001), while M2 macrophages had the strongest negative correlation with plasma cells (*r* = −0.66, *p* < 0.0001) (Figure 15C,D).

In the combined T2DM data set, compared with the healthy group, Monocytes had the lowest degree in the T2DM group (*p* < 0.05) (Figure 15E). Immune score, estimate score and stromal score showed no significant difference (Figure 15F). In the combined OA data set, compared with the healthy group, resting CD4 memory T cells (*p* < 0.05), follicular helper T cells (*p* < 0.001), M2 macrophages (*p* < 0.05) and activated Mast cells (*p* < 0.001) had the lowest degree in the OA group (*p* < 0.05), while resting Mast cells (*p* < 0.001) had the highest degree in the OA group (Figure 15G). Stromal score was higher in the OA group, but there was no significant difference in immune score and estimate score (Figure 15H). In summary, T2DM and OA exhibited distinct levels of infiltration by various immune cell populations, highlighting their potential as pivotal regulatory targets for the management of both conditions.

#### 3.1.6 Transcription regulation analysis and potential chemical prediction

**FIGURE 16:**
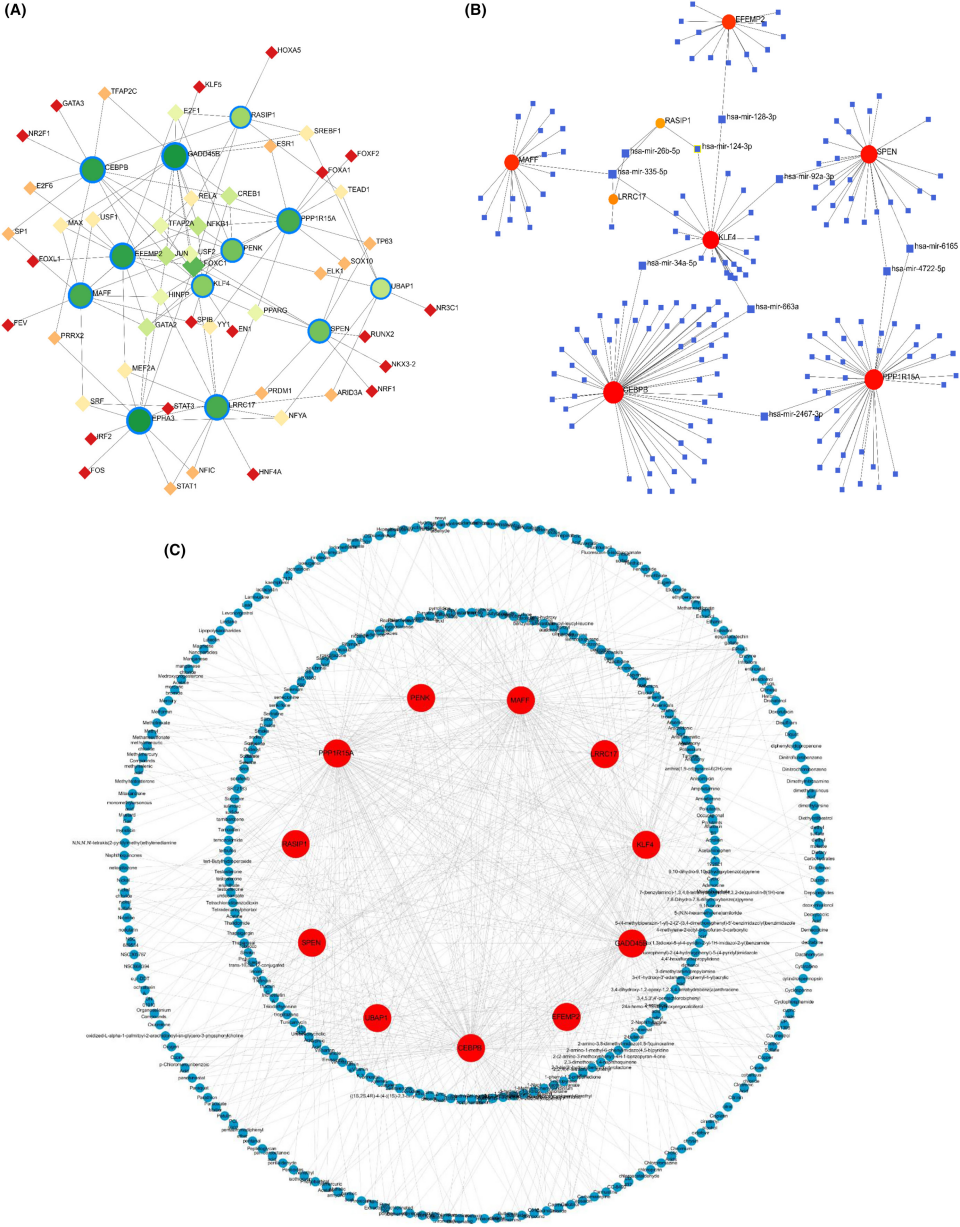
Transcription regulation analysis and potential chemical prediction. (A) The shared genes‐TFs regulatory interaction network. Green circles represent the shared genes, and other receivable diamonds represent TFs. (B) The shared genes‐miRNAs regulatory interaction network. Red and orange circles represent the shared genes, and blue diamonds represent miRNAs. (C) The shared genes‐chemicals regulatory interaction network. Red circles represent the shared genes, and blue circles represent chemicals.

In order to reveal the regulatory mechanism of the shared genes, we used NetworkAnalyst to predict and visualize TFs, miRNAs and potential chemicals interacting with the shared genes. Through the analysis of the interaction network, we identified a total of 61 TFs, 180 miRNAs and 332 chemicals involved in regulating the shared genes. Among them, FOXC1, JUN, NFKB1, CREB1 and GATA2 exhibited higher degrees in the TFs‐shared genes interaction network (Figure 16A). Key miRNAs were also identified from the shared genes‐miRNAs interaction network, including hsa‐mir‐335‐5p, hsa‐mir‐26b‐5p, hsa‐mir‐92a‐3p and hsa‐mir‐34a‐5p (Figure 16B). The top 10 chemicals selected based on their Degree values were considered potential candidates for treating T2DM and OA, including quercetin, valproic acid, trichostatin A, formaldehyde, estradiol, zinc, tretinoin, sodium arsenite, cyclosporine and copper sulphate (Figure 16C).

#### 3.1.7 Gene‐disease association

**FIGURE 17:**
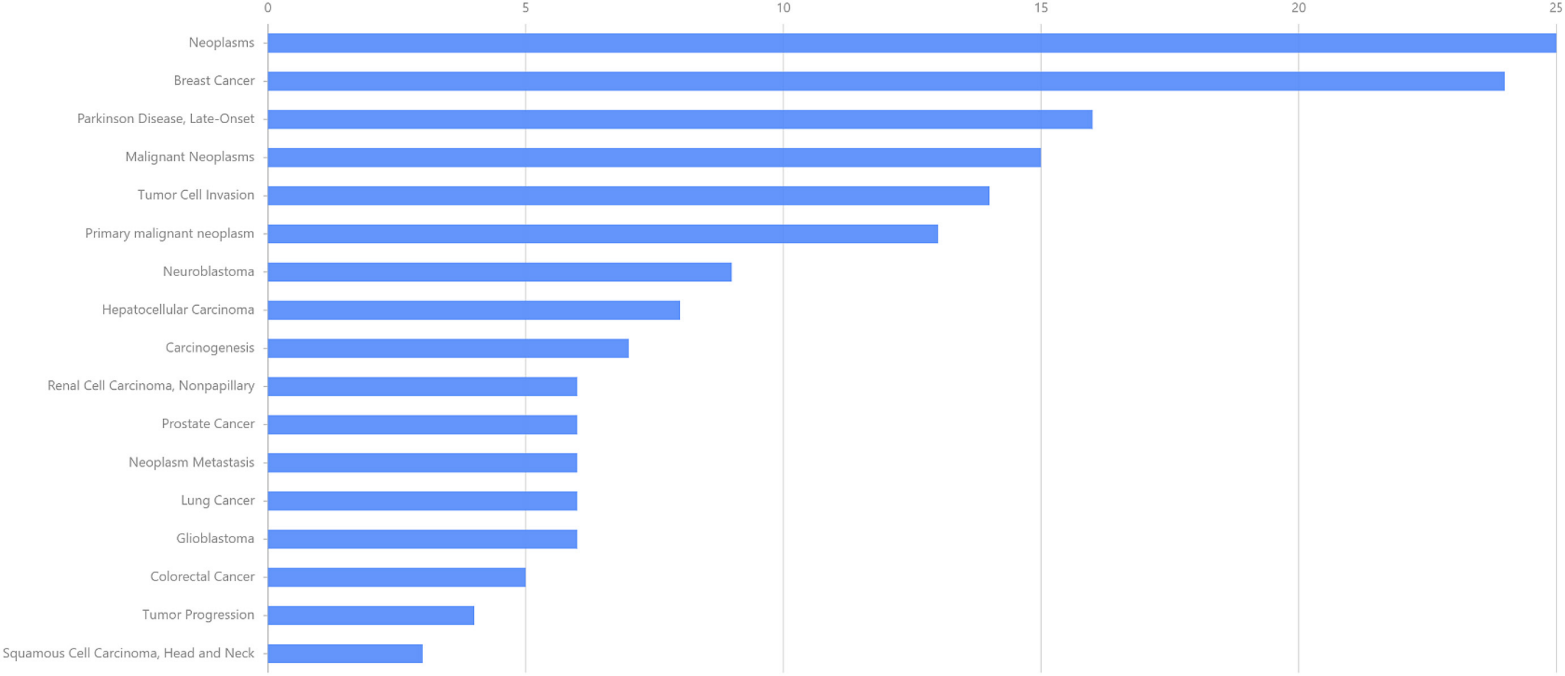
Gene‐disease association. The horizontal axis represents gene abundance, and the vertical axis represents disease names.

By constructing the shared gene‐disease association, we noticed that neoplasms, breast cancer, renal cell carcinoma, nonpapillary, glioblastoma and malignant neoplasms were related to the shared genes (Figure 17). Most of the above diseases are related to the tumour, the tumour is closely related to the immune microenvironment, and the complex and diverse metabolic changes in the immune microenvironment are important factors that directly affect the effect of tumour immunotherapy and drug resistance. The important roles of sugar metabolism, amino acid metabolism, nucleotide metabolism and lipid metabolism in tumour microenvironment are gradually revealed. Targeting these pathways can change tumour immune metabolism and improve the immune microenvironment, thus enhancing the function of immune cells and improving the curative effect of tumour treatment.^23^ Therefore, we suspected that targeting these shared genes can improve the immune microenvironment and play a role in treating diseases.

#### 3.1.8 Relationship between the shared genes and ferroptosis, immunity‐related genes

By correlating the heatmap of shared genes with those associated with ferroptosis and immunity, we have identified that the 12 shared genes actively participate in the regulation of both ferroptosis and immune processes.

**FIGURE 18:**
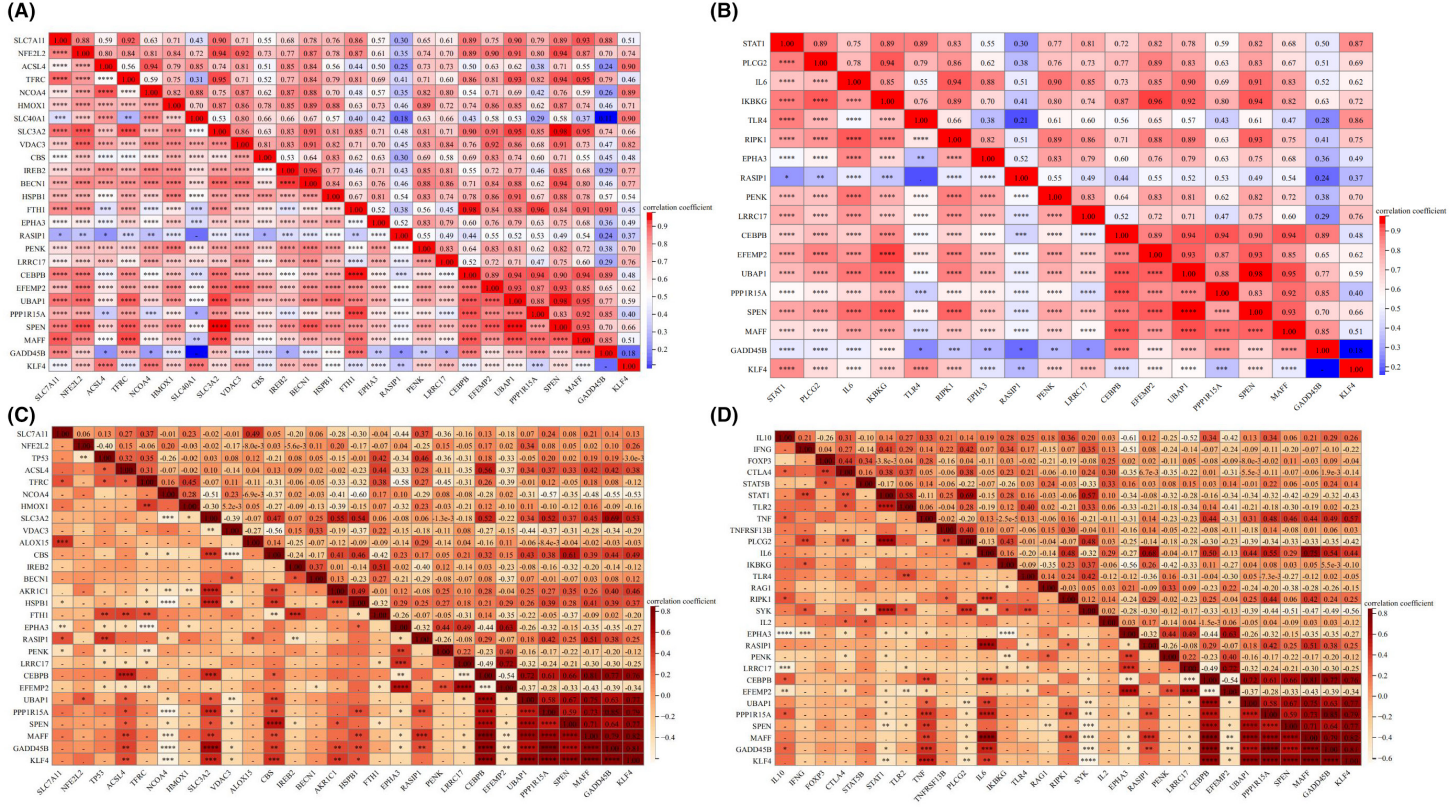
Relationship between the shared genes and the ferroptosis, immunity‐related genes. (A) Heatmap of the shared genes and the ferroptosis‐related genes in the combined T2DM data set. (B) Heatmap of the shared genes and the immunity‐related genes in the combined T2DM data set. (A, B) The redder the colour, the stronger the correlation between the two genes, and the bluer the colour, the weaker the correlation. (C) Heatmap of the shared genes and the ferroptosis‐related genes in the combined OA data set. (D) Heatmap of the shared genes and the immunity‐related genes in the combined OA data set. (C, D) The darker the orange colour, the stronger the correlation between the two genes. *p < 0.05, **p < 0.01, ***p < 0.001, ****p < 0.0001, −, no significant difference.

In the combined DM data set, we found that CEBPB, UBAP1, SPEN and MAFF had a stronger positive correlation with the ferroptosis‐related genes (Figure 18A), while EPHA3, UBAP1 and SPEN had a stronger positive correlation with the immunity‐related genes (Figure 18B). In the combined OA data set, we found that CEBPB, PPP1R15A, SPEN, GADD45B and KLF4 had a stronger positive correlation with the ferroptosis‐related genes (Figure 18C), while CEBPB, EFEMP2, PPP1R15A, MAFF, GADD45B and KLF4 had a stronger positive correlation with the immunity‐related genes (Figure 18D).

#### 3.1.9 ROC analysis

**FIGURE 19:**
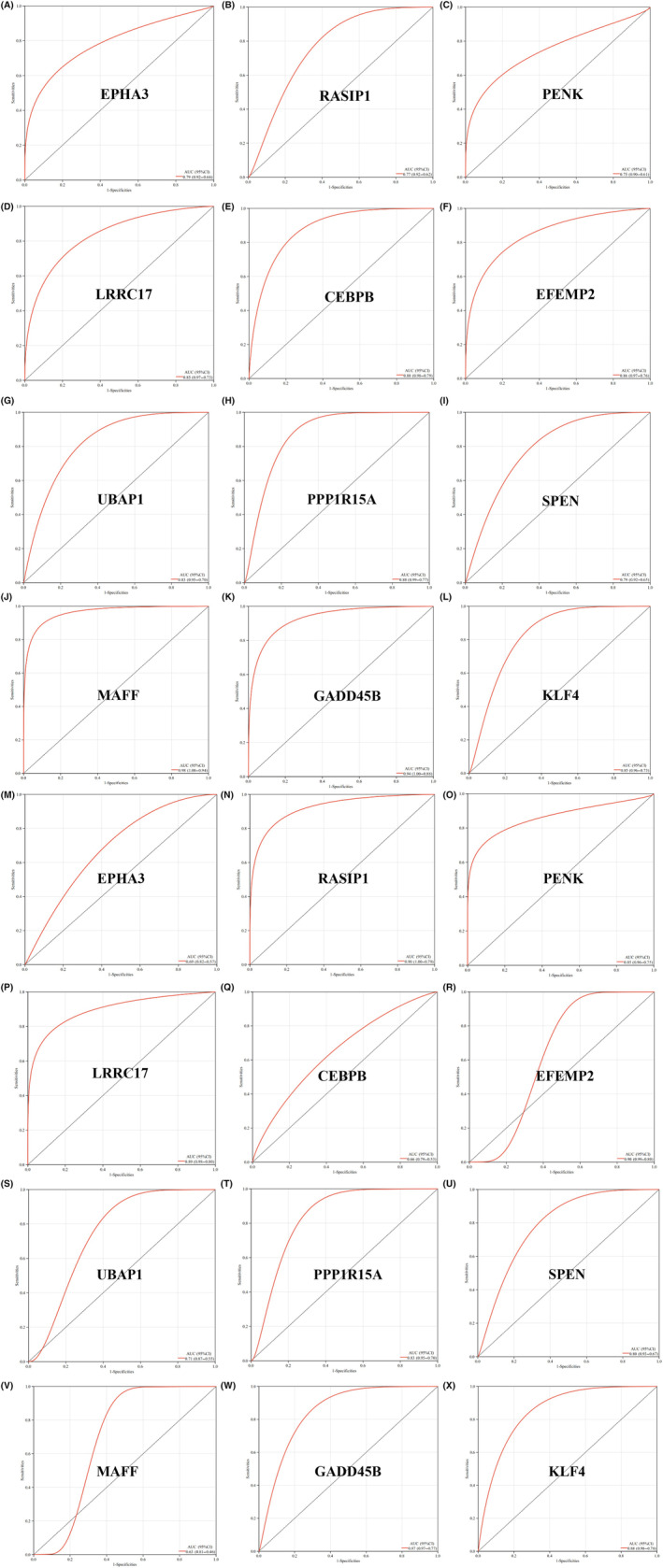
ROC analysis of the shared genes in the combined T2DM and OA data sets. (A) ROC curve of EPHA3 in the combined T2DM data set. (B) ROC curve of RASIP1 in the combined T2DM data set. (C) ROC curve of PENK in the combined T2DM data set. (D) ROC curve of LRRC17 in the combined T2DM data set. (E) ROC curve of CEBPB in the combined T2DM data set. (F) ROC curve of EFEMP2 in the combined T2DM data set. (G) ROC curve of UBAP1 in the combined T2DM data set. (H) ROC curve of PPP1R15A in the combined T2DM data set. (I) ROC curve of SPEN in the combined T2DM data set. (J) ROC curve of MAFF in the combined T2DM data set. (K) ROC curve of GADD45B in the combined T2DM data set. (L) ROC curve of KLF4 in the combined T2DM data set. (M) ROC curve of EPHA3 in the combined OA data set. (N) ROC curve of RASIP1 in the combined OA data set. (O) ROC curve of PENK in the combined OA data set. (P) ROC curve of LRRC17 in the combined OA data set. (Q) ROC curve of CEBPB in the combined OA data set. (R) ROC curve of EFEMP2 in the combined OA data set. (S) ROC curve of UBAP1 in the combined OA data set. (T) ROC curve of PPP1R15A in the combined OA data set. (U) ROC curve of SPEN in the combined OA data set. (V) ROC curve of MAFF in the combined OA data set. (W) ROC curve of GADD45B in the combined OA data set. (X) ROC curve of KLF4 in the combined OA data set.

In order to validate the diagnostic value of the 12 shared genes identified in this study, we constructed ROC curves and calculated their AUC in combined datasets for T2DM and OA. It is evident that MAFF exhibited the highest diagnostic value for T2DM with an AUC of 0.98 (95% CI = 0.94–1.00), while PENK had the lowest diagnostic value for T2DM with an AUC of 0.75 (95% CI = 0.61–0.90) (Figure 19A–L). Similarly, RASIP1 (95% CI = 0.79–1.00) and EFEMP2 (95% CI = 0.80–0.99) demonstrated the highest diagnostic value for OA with identical AUCs of 0.90, whereas MAFF displayed the lowest diagnostic value for OA with an AUC of 0.63 (95% CI = 0.46–0.81) (Figure 19M–X). The fact that all AUC values exceeded 0.5 indicated a high predictive accuracy of these shared genes as potential biomarkers for both T2DM and OA.

#### 3.1.10 Molecular docking

**FIGURE 20:**
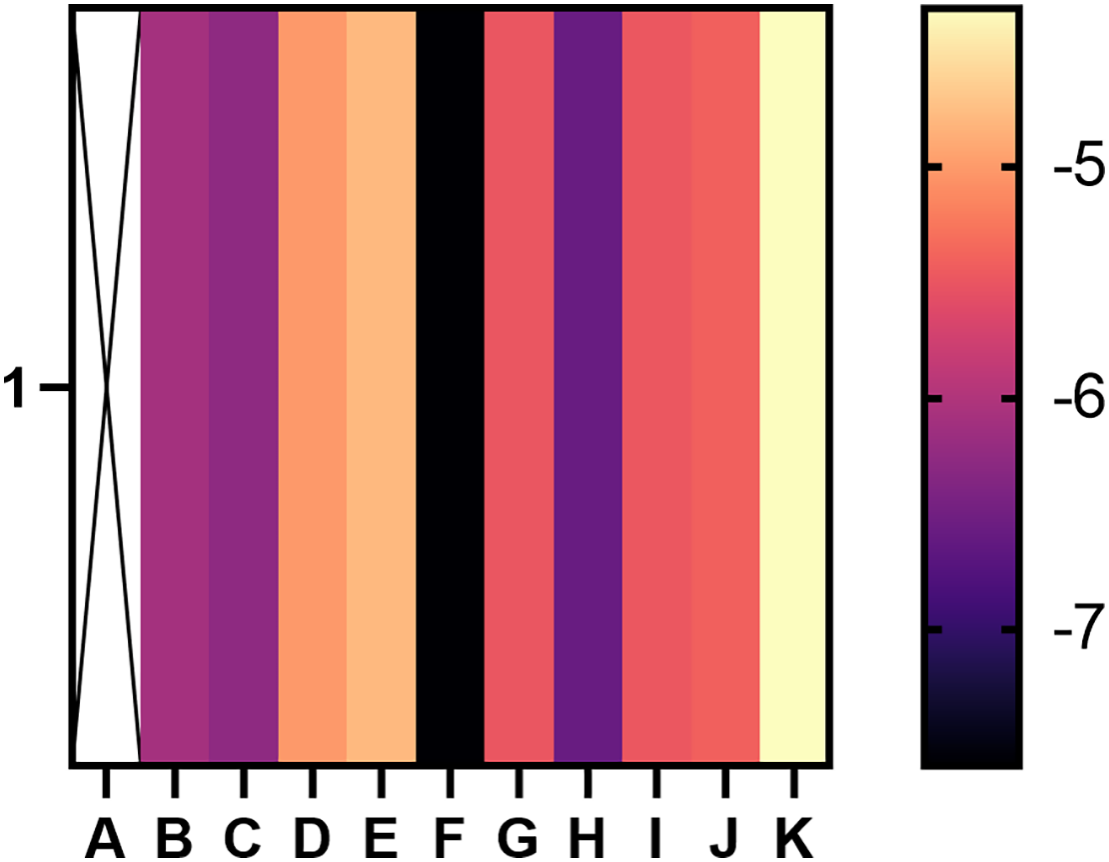
Heatmap of binding affinity. The more purpler the colour is, the smaller stable the binding force is. 1 stands for quercetin, A stands for none, B stands for EPHA3, C stands for RASIP1, D stands for PENK, E stands for CEBPB, F stands for EFEMP2, G stands for UBAP1, H stands for PPP1R15A, I stands for SPEN, J stands for MAFF, and K stands for KLF4.

**FIGURE 21:**
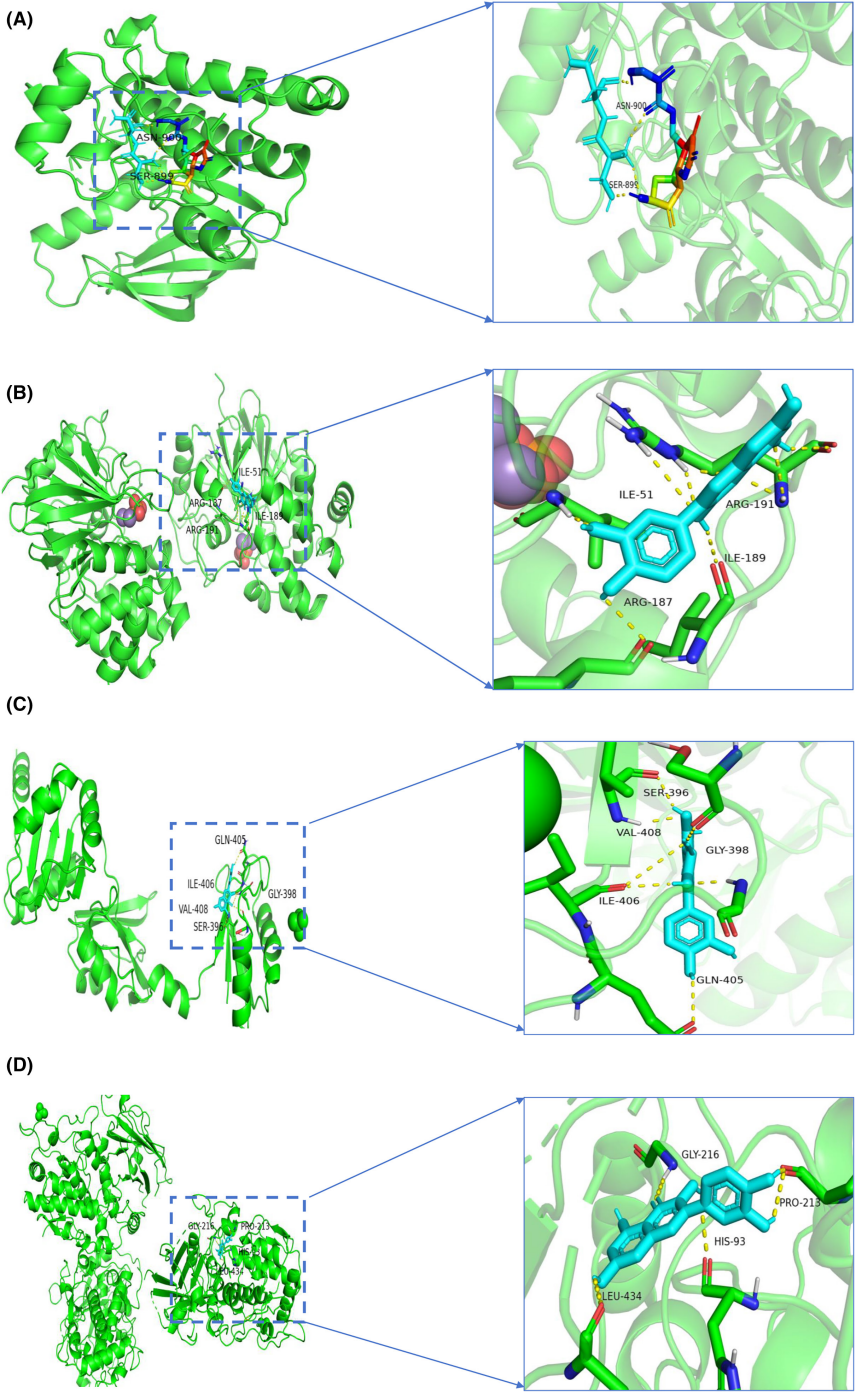
Schematic diagram of docking between quercetin and the four shared genes. (A) Schematic diagram of docking between quercetin and EPHA3. (B) Schematic diagram of docking between quercetin and RASIP1. (C) Schematic diagram of docking between quercetin and EFEMP2. (D) Schematic diagram of docking between quercetin and PPP1R15A.

The docking energy < −4.5 kcal mol^−1^ is considered to have a good binding ability.^24^ The binding energy heatmap of quercetin docking with the 10 shared genes (the molecular structures of LRRC17 and GADD45B are absent in the PDB database) is shown in Figure 20. The more purpler the colour, the smaller the binding energy and the easier it is for quercetin to bind with the shared genes. Among them, the docking binding energy of quercetin and EFEMP2 was the best, reaching −7.59 kcal mol^−1^, which indicated that EFEMP2 may be the first choice target for quercetin to exert its pharmacological activities. We selected the top 4 targets with the most stable binding to quercetin to output visual docking results. The results showed that quercetin formed hydrogen bonds with the hydroxyl groups of ASN‐900 and SER‐899 of EPHA3 (Figure 21A). Quercetin formed π‐π stacking with ILE‐51 and ARG‐187 of RASIP1 and formed hydrogen bonds with ILE‐189 and ARG‐191(Figure 21B). Quercetin formed hydrogen bonds with the hydroxyl groups of SER‐396 and VAL‐408 of EFEMP2 and formed hydrophobic interaction with ILE‐406, GLY‐398 and GLN‐405 (Figure 21C). Quercetin formed π‐π stacking with GLY‐216 and PRO‐213 of PPP1R15A, and formed hydrogen bonds with HIS‐93 and LEU‐434 (Figure 21D). The above findings elucidated the binding patterns and interaction modalities of quercetin with EPHA3, RASIP1, EFEMP2 and PPP1R15A, implying that these proteins may serve as pivotal targets for quercetin in the therapeutic intervention of T2DM and OA.

### 3.2 Bayesian co‐localization analysis

**FIGURE 22:**
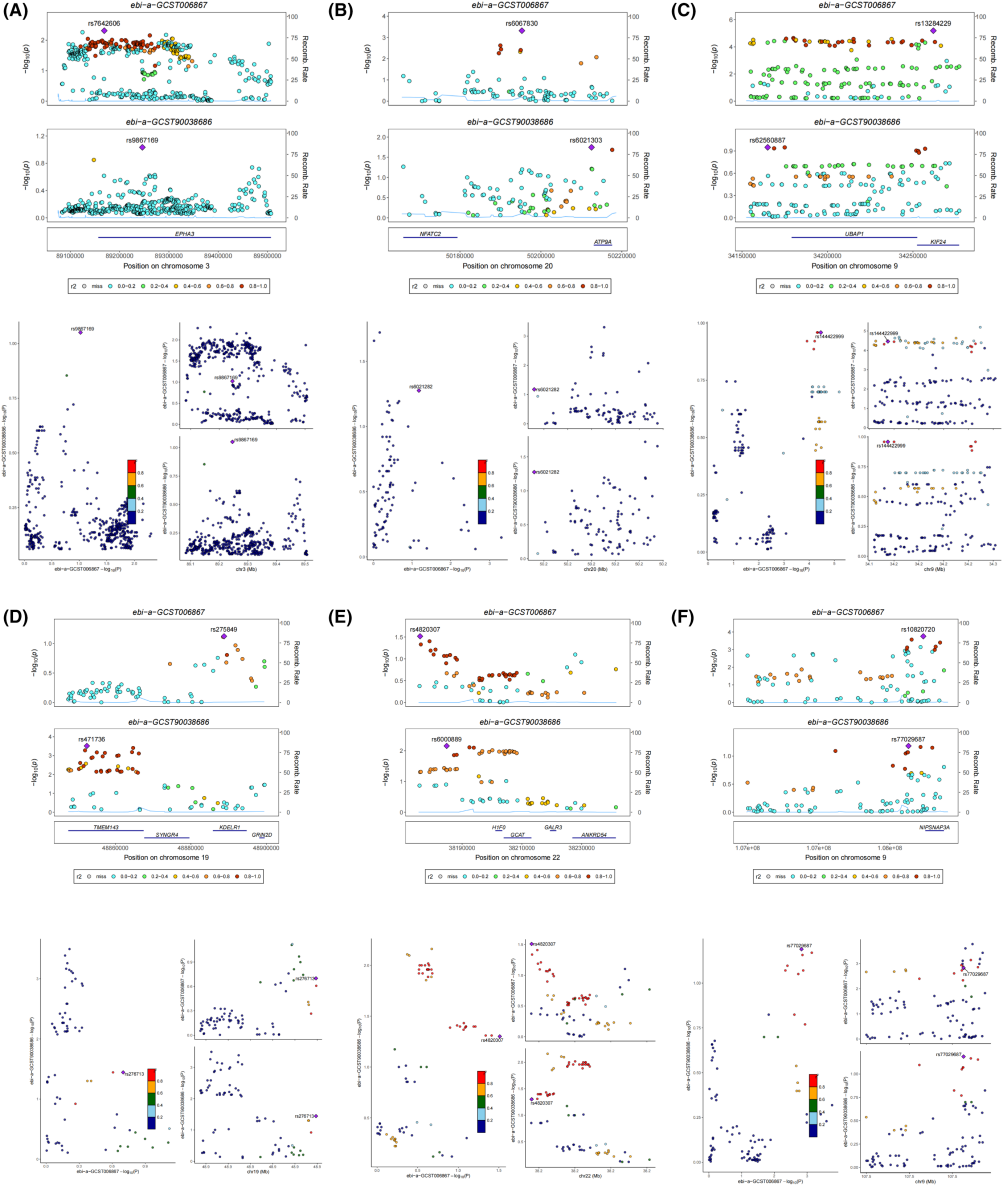
Bayesian co‐localization analysis. (A) Bayesian co‐localization analysis of EPHA3 with T2DM and OA. (B) Bayesian co‐localization analysis of CEBPB with T2DM and OA. (C) Bayesian co‐localization analysis of UBAP1 with T2DM and OA. (D) Bayesian co‐localization analysis of PPP1R15A with T2DM and OA. (E) Bayesian co‐localization analysis of MAFF with T2DM and OA. (F) Bayesian co‐localization analysis of KLF4 with T2DM and OA. The *r* ^2^ value indicates the linkage disequilibrium (LD) between the variants and the top SNPs.

In the GWAS co‐location of EPHA3 with T2DM and OA, rs7642606 and rs9867169 are classified as coloc co‐location in T2DM and OA, both of them are located on chromosome 3 (Figure 22A). In the GWAS co‐location of CEBPB with T2DM and OA, rs6067830 and rs6021313 are classified as coloc co‐location in T2DM and OA, both of them are located on chromosome 20 (Figure 22B). In the GWAS co‐location of UBAP1 with T2DM and OA, rs13284229 and rs62560887 are classified as coloc co‐location in T2DM and OA, both of them are located on chromosome 9 (Figure 22C). In the GWAS co‐location of PPP1R15A with T2DM and OA, rs275849 and rs411736 are classified as coloc co‐location in T2DM and OA, both of them are located on chromosome 19 (Figure 22D). In the GWAS co‐location of MAFF with T2DM and OA, rs4820307 and rs6000889 are classified as coloc co‐location in T2DM and OA, both of them are located on chromosome 22 (Figure 22E). In the GWAS co‐location of KLF4 with T2DM and OA, rs10820720 and rs77029687 are classified as coloc co‐location in T2DM and OA, both of them are located on chromosome 9 (Figure 22F). There was no result of GWAS co‐location of GADD45B with T2DM and OA. However, their PPH4 values are all less than 0.8 (Table S3), indicating that the seven shared genes have no genetic correlation with T2DM and OA, while observational studies showed that the seven shared genes were involved in the pathogenesis of T2DM and OA. We thought that observational studies may be influenced by acquired confounding factors, such as environmental factors and acquired genetic variation.

## 4 DISCUSSION

T2DM is closely associated with the pathogenesis and progression of OA, wherein hyperglycaemia and insulin resistance play pivotal roles in inducing OA in individuals with T2DM. Hyperglycaemia exerts its influence on articular cartilage, synovium and subchondral bone, thereby promoting the development of OA. Insulin resistance impedes the binding between insulin and its receptors, consequently diminishing the protective effect of insulin on joints and further facilitating the advancement of OA. Nevertheless, a comprehensive understanding regarding the intricate interplay between T2DM and OA remains elusive at present. Henceforth, it holds immense significance to investigate the underlying comorbidity mechanisms linking T2DM with OA while evaluating quercetin's therapeutic potential for early intervention and treatment of these two diseases.

In this study, four microarray data sets of T2DM and OA were analysed by three bioinformatics methods, and 12 shared genes were obtained finally, including EPHA3, RASIP1, PENK, LRRC17, CEBPB, EFEMP2, UBAP1, PPP1R15A, SPEN, MAFF, GADD45B and KLF4. The verification of the shared genes in the clinical database and the ROC curves proved that the shared genes had a promising predictive performance of T2DM and OA. Among them, seven genes (EPHA3, CEBPB, UBAP1, PPP1R15A, MAFF, GADD45B and KLF4) have been reported to be related to the pathological mechanism of T2DM and OA. EPHA3 has been proven to play an important role in cancer^25^ and energy metabolism,^26^ and it might participate in the currency of OA.^27^ The expression of CEBPB decreased in patients with DN,^28^ and the overexpression of CEBPB could reverse the proliferation of OA‐FLS cells induced by hypoxia.^29^ UBAP1 played an important role in NF‐kB signal transduction.^30^ It is reported that PPP1R15A inactivation could increase insulin resistance^31^ and play a proinflammatory role through endoplasmic reticulum stress.^32^ The decrease of MAFF can inhibit the expression of antioxidant genes and increase the apoptosis of β cells induced by oxidative stress,^33^ while the overexpression of MAFF can inhibit the transcription of NF‐E2‐related factors, which leads to immune and inflammatory reactions.^34^ GADD45B can be used as a prognostic biomarker in the diagnosis of DN,^35^ and it has been proved that the expression of GADD45B is downregulated in synovial tissue of OA.^36^ KLF4 can inhibit inflammation and bone destruction in OA,^37^ and inhibition of KLF4 expression can promote the conduction of the NF‐kB signal pathway and then improve the insulin resistance of macrophages.^38^ Two genes (RASIP1 and PENK) have been reported to be related to T2DM. RASIP1 is a vascular regulator and has been proven to be a risk factor for DM.^39^ The increase of PENK level was related to cardiovascular mortality in patients with T2DM.^40^ Two genes (EFEMP2 and LRRC17) have been reported to be related to OA. LRRC17 was reported to regulate bone metabolism through the Wnt signalling pathway to prevent osteoporosis.^41^ EFEMP2 is the downstream negative regulatory target of miR‐211‐5p, which was involved in chondrocyte differentiation and can reduce the expression level of proinflammatory cytokines.^42^ At present, there is no literature report on the relationship between SPEN and OA and DM.

Functional enrichment analysis revealed that the shared genes were predominantly enriched in signalling pathways associated with p53, IL‐17, NF‐kB and MAPK. It has been found that p53 participates in the occurrence and development of insulin resistance through various channels. In islet cells, after p53 is activated, miR34a in islet cells is increased and the expression of vesicular‐associated membrane protein 2, a key protein involved in exocytosis in β cells, is inhibited, which in turn affects the normal secretion and release of insulin in islet cells. At the same time, p53 can also activate various microRNA to induce apoptosis of islet cells through the expression of pro‐apoptosis genes.^43^ The increase of p53 level can promote the pathological process of OA.^44^ IL‐17 and NF‐kB signalling pathways are inflammation‐related pathways, hyperglycaemia caused by T2DM will aggravate the oxidative stress of cells and increase the synthesis of proinflammatory cytokines. In addition, a chronic high glucose environment makes articular chondrocytes more sensitive to proinflammatory factors.^45^ The results indicated that the aforementioned signalling pathways are implicated in the common pathogenesis of both T2DM and OA. GSEA results demonstrated a close association between the shared genes high subtype and DM, inflammatory response and immune infiltration. Therefore, we hypothesized that altering the immune microenvironment may be a potential therapeutic approach for treating T2DM and OA.

In order to elucidate the potential mechanistic relationship between ferroptosis, immunity and the two diseases, we conducted an analysis on the correlation between the shared genes and the genes associated with ferroptosis and immunity. The findings revealed varying degrees of association between the shared genes and both ferroptosis‐related and immunity‐related genes, suggesting that these genetic factors may play crucial roles in mediating the comorbidity of T2DM and OA. Furthermore, it is plausible that there exists a crosstalk between ferroptosis and immunity in T2DM and OA.

We also analysed the gene regulation of the shared genes and found that they can interact with various miRNAs, and TFs to regulate the occurrence and development of T2DM and OA. The possible targeted drugs were predicted, which will provide a broad prospect for future drug development. Additionally, we assessed the interaction between the predicted hub compound quercetin and the common genes. The findings revealed that EPHA3, RASIP1, EFEMP2 and PPP1R15A may serve as pivotal targets of quercetin in the treatment of T2DM and OA. This suggested that quercetin holds potential as a therapeutic agent for T2DM and OA by selectively targeting these shared genes.

In this study, we employed multiple bioinformatics approaches to enhance our understanding of the relationship between T2DM and OA. Although previous studies have discussed the hub genes related to T2DM or OA, respectively, the research on exploring the common molecular mechanism between T2DM and OA by bioinformatics methods is still blank. We conducted analyses of potential therapeutic targets, mechanisms and drugs associated with both conditions. Furthermore, we explored the correlation between shared genes and immunity as well as ferroptosis‐related genes, suggesting a possible crosstalk between immune cells and ferroptosis in T2DM and OA. Based on our findings, we proposed that targeting the 12 identified shared genes could be a promising approach for treating T2DM and OA by modulating the p53, IL‐17, NF‐kB and MAPK signalling pathways while regulating ferroptosis and immunity.

Our study screened the shared genes based on the three different bioinformatics methods, and this is the first study to explain the comorbidity mechanism of T2DM and OA. However, there are still some limitations in our study. Firstly, all the results were derived from open databases, future investigations should aim to verify the reliability of the shared genes as biomarkers for T2DM and OA through experimental verification in vitro and *vivo*. Secondly, the specific mechanism by which these shared genes induce ferroptosis in T2DM and OA, subsequently regulating immunity, remains unclear. This aspect holds equal importance for us. Thirdly, it is essential to elucidate the precise mode of action of Quercetin in treating T2DM and OA. Future molecular dynamics simulation should be applied in this study. Fourthly, the progression from T2DM to OA is a dynamic and gradual process. In future studies, we will investigate the gene matrix of T2DM complicated with OA to identify marker genes associated with this process. Fifthly, additional computational models such as gene function and protein association (GFPA) model,^46^ graph convolutional neural network and conditional random field (GCNCRF) model,^15^ deep learning model based on capsule network and attention mechanism (DCAMCP),^47^ and learning model based on auto‐encoder and non‐negative matrix factorization (MDA‐AENMF)^48^ should be employed for predicting associations between gene function and cell surface proteins, lncRNA–miRNA interactions, drug design, as well as associations between metabolites and diseases, respectively, these aspects will be explored in our future research endeavours. Finally, although the study provide valuable insights into the pathological mechanisms of T2DM and OA. However, conducting an in‐depth exploration of the gene/protein signalling network holds immense significance in advancing our understanding of regulatory mechanisms and identifying potential therapeutic targets for diseases. Therefore, functional determination is indispensable in verifying these potential therapeutic targets. By referring to ODE‐based theoretical models such as the SWATH‐MS‐based network model,^49^ mRNA‐driven protein liquid–liquid phase separation model^50^ and ‘seesaw model’,^51^ we can validate our research findings. These transformative discoveries are crucial for bridging the gap between preclinical research and clinical application, ultimately guiding the development of targeted treatment interventions for patients with T2DM and OA.

## 5 CONCLUSION

In this study, we identified 12 shared genes (EPHA3, RASIP1, PENK, LRRC17, CEBPB, EFEMP2, UBAP1, PPP1R15A, SPEN, MAFF, GADD45B and KLF4) and investigated the underlying comorbidity mechanism between T2DM and OA. Furthermore, we proposed a potential crosstalk between ferroptosis and immunity in these two diseases. Additionally, targeting the shared genes with quercetin holds promise as a therapeutic approach for both T2DM and OA. These findings provide valuable insights into the pathological mechanisms of T2DM and OA.

## AUTHOR CONTRIBUTIONS

**Siyuan Song:** Conceptualization (equal); resources (equal). **Jiangyi Yu:** Conceptualization (equal); software (equal).

## FUNDING INFORMATION

This study was supported by the National Natural Science Foundation of China (82174293 and 82374355).

## CONFLICT OF INTEREST STATEMENT

The authors declare that they have no competing interests.

## Supporting information


Figure S1

Table S1

Table S2

Table S3

## Data Availability

The data sets presented in this study can be found in online repositories. The names of the repository and accession number(s) can be found within the article.
