# Supplementary figures and images for: Identification of the shared genes in type 2 diabetes mellitus and osteoarthritis and the role of quercetin
Source: J Cell Mol Med. 2024 Feb 8;28(4):e18127. doi: 10.1111/jcmm.18127 (PMC10853600; doi:10.1111/jcmm.18127)

**Supplementary Figure 1**

**A**

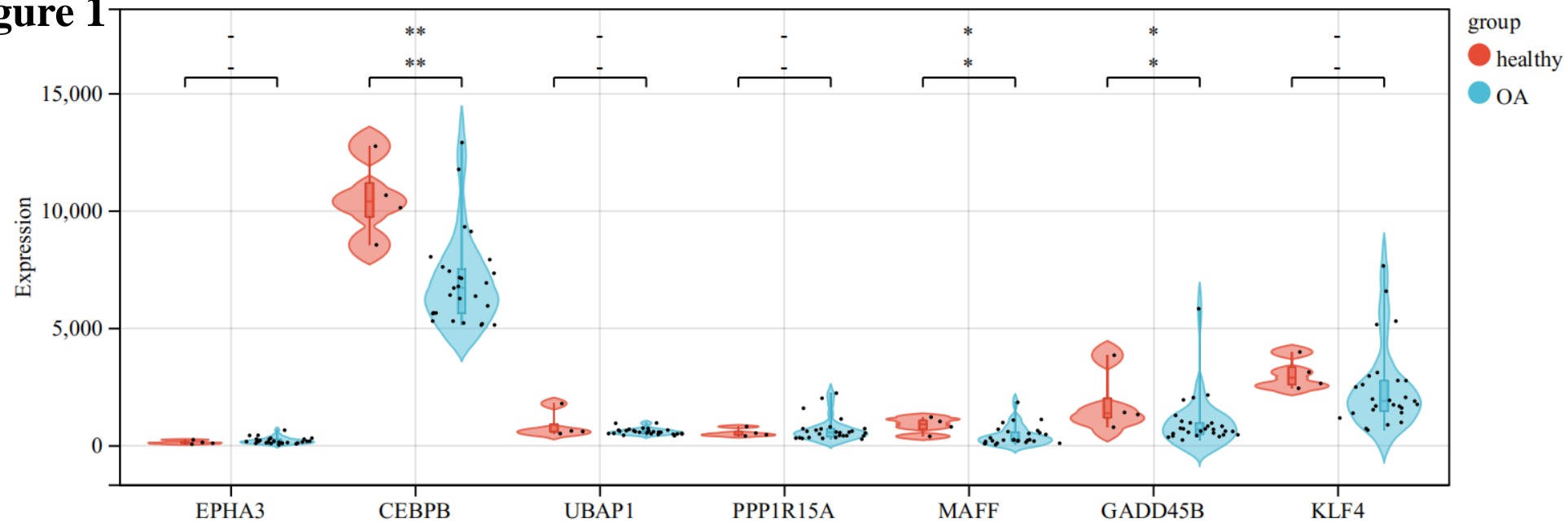

**B**

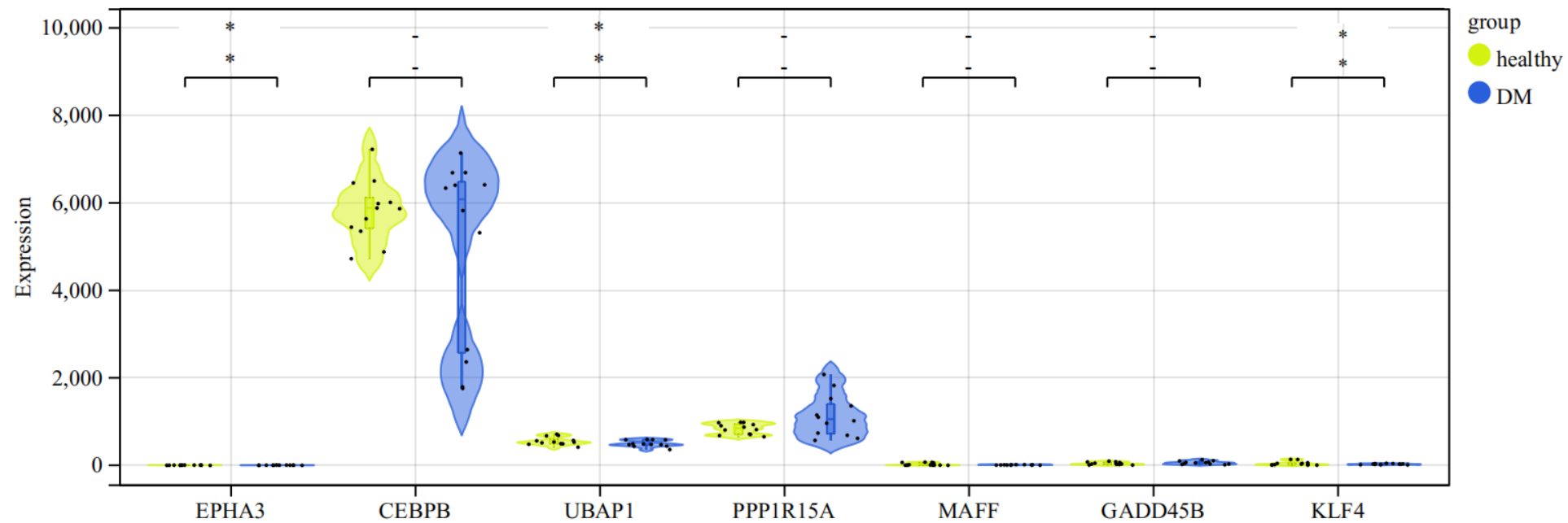

Supplement: Supplementary file 1 — Figure S1 [file JCMM-28-e18127-s004.pdf]
